# Integrating ecological and recreational functions to optimize ecological security pattern in Fuzhou City

**DOI:** 10.1038/s41598-024-84660-1

**Published:** 2025-01-04

**Authors:** Junting Bai, Rui Sun, Yifan Liu, Jie Chen, Xiaohe Li

**Affiliations:** 1 College of Landscape Architecture and Art, Fujian Agriculture and Forestry University, Fuzhou, 350002 China; 2 Strait Beautiful Rural Human Settlement Environment Research Center, Fuzhou, 350002 China

**Keywords:** Ecological security pattern, Recreational flow, Fuzhou City, Morphological spatial pattern analysis model, Circuit theory, Ecology, Environmental sciences

## Abstract

The scientific establishment of the Ecological Security Pattern (ESP) is crucial for fostering the synergistic development of ecological and recreational functions, thereby enhancing urban ecological protection, recreational development, and sustainable growth. This study aimed to propose a novel method of constructing ESP considering both ecological and recreational functions, and to reconstruct ESP by weighing the relationship between ecological protection and recreational development. Utilizing Fuzhou City as a case study, a comprehensive application of methodologies including Morphological Spatial Pattern Analysis (MSPA), landscape connectivity analysis, ArcGIS spatial analysis, social network analysis (SNA), and circuit theory is employed to develop both the ESP and the Recreational Spatial Pattern (RSP). A trade-off matrix is created to facilitate the reconstruction of the ESP, delineate functional zones, and identify strategic points, followed by a thorough optimization and development strategy. The analysis revealed 36 ecological source areas (ESAs) within Fuzhou City, encompassing a total area of 5807.90 km², predominantly situated in the western and northern regions. Additionally, 98 ecological corridors (ECs) were identified, extending over 2500.55 km, alongside 100 ecological pinch points (EPPs) and 146 ecological barrier points (EBPs). The city also contains 57 recreational nodes (RNs),, which display a spatial distribution characterized by a “dense in the east, sparse in the west” pattern. Furthermore, 165 recreational corridors (RCs) were extracted, covering a distance of 3795.21 km. Based on the trade-off matrix, Fuzhou City was categorized into eight functional zones: ecological core zone, ecological important zone, eco-recreation key trade-off zone, eco-recreation secondary trade-off zone, recreational core zone, recreational important zone, recreational development zone, and elastic development zone.The study identified 95 key strategic points and 475 sub-strategic. A multifunctional and complex ESP was constructed, characterized by “one core, five districts, six corridors, and seven wedges”, and a tailored ecological and recreational planning and development strategy for Fuzhou City was proposed. This research contributes a theoretical framework for the construction and optimization of a multifunctional ESP and supports the coordinated high-quality development of ecological protection and recreational activities in urban environments.

## Introduction

The rapid and extensive expansion of urban area, alongside the increasing diversity and complexity of socio-economic activities, has precipitated significant alterations in land use patterns^1,2^. These transformations have led to a notable reduction and fragmentation of ecological spaces, diminished ecosystem stability, and weakened landscape connectivity within urban environments^3^. The Ecological Security Pattern (ESP) represents a potential spatial configuration model that is developed by identifying and delineating critical elements and their strategic locations within natural landscapes^4^. This model serves as a fundamental framework for the sustainable and stable provision of ecosystem services^5^. As a pivota strategy for urban renewal and rural planning, ESP is instrumental in maintaining ecological security, fostering biodiversity conservation, and mitigating landscape degradation^6^. It is widely acknowledged as essential for ensuring the sustainable development of human societies and facilitating harmonious coexistence with the ecological environment^4^. In light of ongoing global population growth and heightened public awareness regarding the health benefits and quality of life improvements associated with natural recreation, urban residents and tourists are increasingly prioritizing and exploring diverse recreational opportunities^7^. Green spaces, including parks, forests, lakes, and semi-natural areas, act as conduits for fostering close connections with nature, enhancing physical and mental well-being, and achieving a work-life balance^8^. However, the growing spatial and temporal overlap, as well as the incompatibility of recreational behaviors and objectives, combined with rising participation rates and the diversification of recreational activities, have intensified conflicts and supply-demand imbalances between recreational needs and ecological integrity^9^. These trends exacerbate the contradictions in the supply and demand for recreational resources within ecological spaces, resulting in a decline in the quality of urban recreational environments and a decrease in resident satisfaction^10^. In response to these challenges, China has recognized the optimizing of land spatial development patterns as a critical measure in the pursuit of ecological civilization to address severe ecological issues^11^. The “13th Five-Year Plan for Ecological and Environmental Protection” has proposed the establishment of an ecological public service network, with the aim of enhancing public service facilities for ecological spaces and diversifying and improving the supply of ecological services, including recreation and education^12^. Consequently, the escalating conflict between urban ESP and the demands of recreational development necessitates scientific planning and effective management to achieve coordination and balance, thereby ensuring urban health and sustainable development^7,13^.

The ESP represents a potential spatial configuration that encompasses essential elements such as points, lines, and locations within a given landscape, along with their interrelated spatial relationships^14^. This framework has emerged as a vital spatial strategy aimed at reconciling the inherent conflicts between ecological protection and economic development^15^. By synthesizing various components, ESP effectively integrates spatial patterns with ecological processes, thereby establishing a sustainable landscape framework that yields substantive environmental benefits^16^. The construction of ESP is primarily involves three research steps: the identification of ecological source areas (ESAs) and ecological source points (ESPs), the creation of ecological resistance surface (ERS), and the extraction of ecological corridors (ECs)^17^. ESAs are critical zones that are fundamental to the essential functions and the overall integrity of ecosystems^18^. Early studies primarily used direct identification methods, which involved selecting extensive areas such as forests, grasslands, nature reserves, and watersheds as ESAs^19^. However, this approach was ofen subjective and did not adequately account for environmental heterogeneity and landscape connectivity^20^. Some investigations have utilized quantitative evaluations of ecosystem services to delineate ESAs^21^. Nevertheless, these studies lacked systematic analysis of the structural characteristics inherent to these areas^22^. Morphological Spatial Pattern Analysis (MSPA) has proven effective in identifying and distinguishing various landscape types, highlighting structural connections, and quantifying landscape complexity and dynamics, thereby enhancing the scientific rigor associated with ESAs identification^23^. Consequently, MSPA has become a widely adopted method for identifying ESAs and provides a significant methodological reference for the present study^16^. The objective of constructing the ERS is to quantify and represent the various obstacles that information and species must navigate as they disperse from source locations within the natural environment^24^. The predominant approach to developing the ERS involves systematically defining different levels of resistance based on influencing factors and their respective degrees of impact. Although the ERS constructed using this method is more systematic and objective, scholars have primarily focused on the ecological processes of natural elements within the network, while paying less attention to the spatial connections and interactions between natural ECs and recreational networks. Furthermore, recreational factors have not been adequately considered, and there is a lack of an evaluation system based on recreational factors^16^, making it difficult to systematically and comprehensively reflect the integrated functions and service values of ecological networks. Therefore, it is essential to conduct a comprehensive analysis of recreational resources, roads, infrastructure, and other recreational factors to construct a recreational resistance surface (RRS). The third step in constructing ESP is the extraction of ECs. Recent research has utilized the Minimum Cumulative Resistance (MCR) model to compute optimal pathways for ECs. This model quantifies the energy expenditure associated with species migration from source to target habitats, while also simulating various ecological flow patterns. Although the MCR model is effective in pinpointing optimal migration routes, it exhibits certain limitations in delineating specific spatial boundaries for ECs and in identifying key nodes^25^. Conversely, circuit theory not only simulates the direction of biological flow within ecosystems but also identifies key nodes and areas within the ecological network using analogies to electrical current intensity. This approach effectively addresses the limitations of the MCR model^26^. Circuit theory has been extensively utilized in research concerning multifunctional corridors, including scenic byways^27^, heat island ventilation corridors^28^, and wildlife migration pathways^29^. Nevertheless, the existing body of research concerning the development of recreational corridors (RCs) remains inadequate, resulting in a deficiency of systematic and scientifically grounded methodologies for their construction. This shortcoming complicates the effective reconciliation of ecological conservation with recreational utilization in practical implementations. Therefore, based on circuit theory and following the methodology for constructing ECs, this study builds a RRS and identifies RCs using the Linkage Mapper tool. Overall, current research on ESP focuses primarily on ecological functions, frequently neglecting the significance of recreational functions. As urbanization continues to advance, it is imperative to simulate ESP within various functional environments to effectively tackle the intricate challenges encountered in urban settings and to foster sustainable urban development^11^. While some scholars have constructed ecological networks from a multifunctional perspective, integrating “source-sink” processes^30^ and evaluating core areas through multi-scenario quality models^11^, these studies typically focus on isolated components, such as ESAs or ECs. This approach often neglect the comprehensive consideration and interaction of multiple elements and functions, which may result in potential conflicts within functional spatial layouts. Therefore, this study aims to incorporate recreational factors into the construction of the ESP to achieve a dual functionality of both ecology and recreation. The inclusion of this coupled relationship not only enhances the practicality of the ESP but also better meets the diverse needs of residents and visitors. Zhang et al. proposed a trade-off matrix that incorporates dimensions of “biological-recreational-management” and utilized it to the management of outdoor recreational opportunities^31^. Subsequently, Liu et al. proposed a trade-off matrix framework based on ecological and recreational functions. This framework effectively delineates the trade-off relationships between these two functions, identifies areas with significant trade-off issues, and demonstrates considerable operational applicability^30^. Current studies has yet to explore the application of trade-off matrices in the optimization of ESP, resulting in a notable absence of systematic evaluations regarding the trade-off dynamics between urban ecological functions and recreational functions. Building upon the construction of ecological and recreational corridors, this study uses a trade-off matrix to model the spatial distribution of ecological and recreational functions, assessing the trade-off relationships between these functions. It distinguishes the construction focus and priorities for different functional areas, identifies regions where trade-off issues are most pronounced, reconstructs the ecological security pattern, and develops target planning and management strategies. This approach will facilitate the scientific and refined management of urban ecological and recreational landscape resources, promoting the coordinated development of regional landscape ecology and recreational functions, and optimizing their overall effectiveness.

Recreation serves as one of the four major urban functions and constitutes a significant aspect of cultural ecosystem services, thereby influencing individuals’ quality of life and well-being^32^. Previous research has demonstrated that recreational activities in urban lakes, rivers, forest parks, and leisure farms significantly enhance physical and mental health, alleviate stress, and promote the construction and strengthening of positive social relationships^33,34^. Furthermore, research in recreational ecology indicates that nature-oriented recreational experiences, by strengthening emotional connections with local environments and deepening environmental value recognition, significantly and positively influence ecological protection^32^. Incorporating recreational functions into the ESP framework for Fuzhou City not only enhances ecosystem service functions and improves environmental quality, but also optimizes recreational functions and experiences, thereby yielding multifaceted benefits. The concept of recreational flow, influenced by the movements and choices of tourists, represents a social network structure that accurately delineates the trajectories of individuals or groups within various recreational spaces^35^. It reveals the complex mechanisms of how recreational needs are stimulated, responded to, and satisfied^33^. With the integration of the Internet, communication technologies, and geographic information systems, travel platforms such as “TripAdvisor,” “Mafengwo,” and “Ctrip” have generated and collected extensive user travel data and geotagged photos. These travel logs serve as a crucial data foundation for analyzing users’ spatiotemporal footprints and preferences^36^. In this context, Social Network Analysis (SNA), utilizing tools such as Ucinet and Gephi, is widely employed to investigate the spatial structure of recreational flow^35^, recreational experiences^37^, and the construction of destination systems^38^. By revealing the network structure of recreational flows and the roles and statuses of nodes, SNA provides essential data support and theoretical basis for urban planning, tourism resource development and conservation, as well as public policy formulation^36^. This study integrates SNA principles with diverse datasets to explore the spatiotemporal characteristics of the alignment between recreational resource supply and demand^39^, as well as the scientific construction of corridor network systems^40^, thereby opening new research perspectives and pathways. However, the application of this method in the field of ecological protection is quite limited, which is not conducive to a comprehensive analysis of the multidimensional interactions between ecology and recreation, among other factors. Therefore, this study introduces the principles of SNA, considering the spatial distribution of points of interest and digital footprints, as well as their impact on recreational activities. By integrating recreational flow analysis, recreational nodes are selected. Subsequently, the RCs is developed by combining the RRS with circuit theory, aiming to enhance the accuracy and practicality of the RSP construction.

Fuzhou City, recognized as a new international gateway and a high-quality development within Fujian Province, serves as a crucial hub that connects the northern and southerncoastal regions while linking inland areas to international markets. This positioning is vital for the implementation of regional strategies aimed at coordinated multifunctional development in Fujian. Nevertheless, amidst rapid urbanization, Fuzhou is confronted with a range of urban challenges, including fragmented living environments, diminished connectivity along corridors, and a lack of adequate recreational facilities^41^. Consequently, there is an urgent necessity to establish an integrated multifunctional ecological security pattern that reconciles the demands of swift urbanization with ecological sustainability. The city’s varied topography, which encompasses mountains, hills, plains, and basins, in conjunction with its abundant natural and cultural recreational resources, renders it an exemplary case for investigating composite ecological security patterns. This study is thus centered on Fuzhou City, aiming to identify efficient and sustainable strategies for optimizing the ESP while harmonizing recreational development with ecosystem considerations. The primary objectives of this research are as follows: (1) To construct the ESP by identifying ESAs, developing and categorizing ECs, and pinpointing ecological pinch points (EPPs) and ecological barrier points (EBPs) through the application of the MSPA model, landscape connectivity analysis, and circuit theory. (2) To formulate the recreational spatial pattern (RSP) by identifying recreational nodes (RNs) and corridors utilizing ArcGIS spatial analysis, SNA, and circuit theory. (3) To reconstruct the ESP of Fuzhou City by employing the trade-off matrix method to evaluate the relative significance of ecological and recreational functions across various units, thereby identifying functional zones and strategic points. (4) To propose planning, construction, and management strategies informed by the reconstructed ESP, thereby offering guidance for the optimization of multifunctional landscape patterns in Fuzhou and comparable urban environments.

## Materials and methods

### Study area

**Fig. 1:**
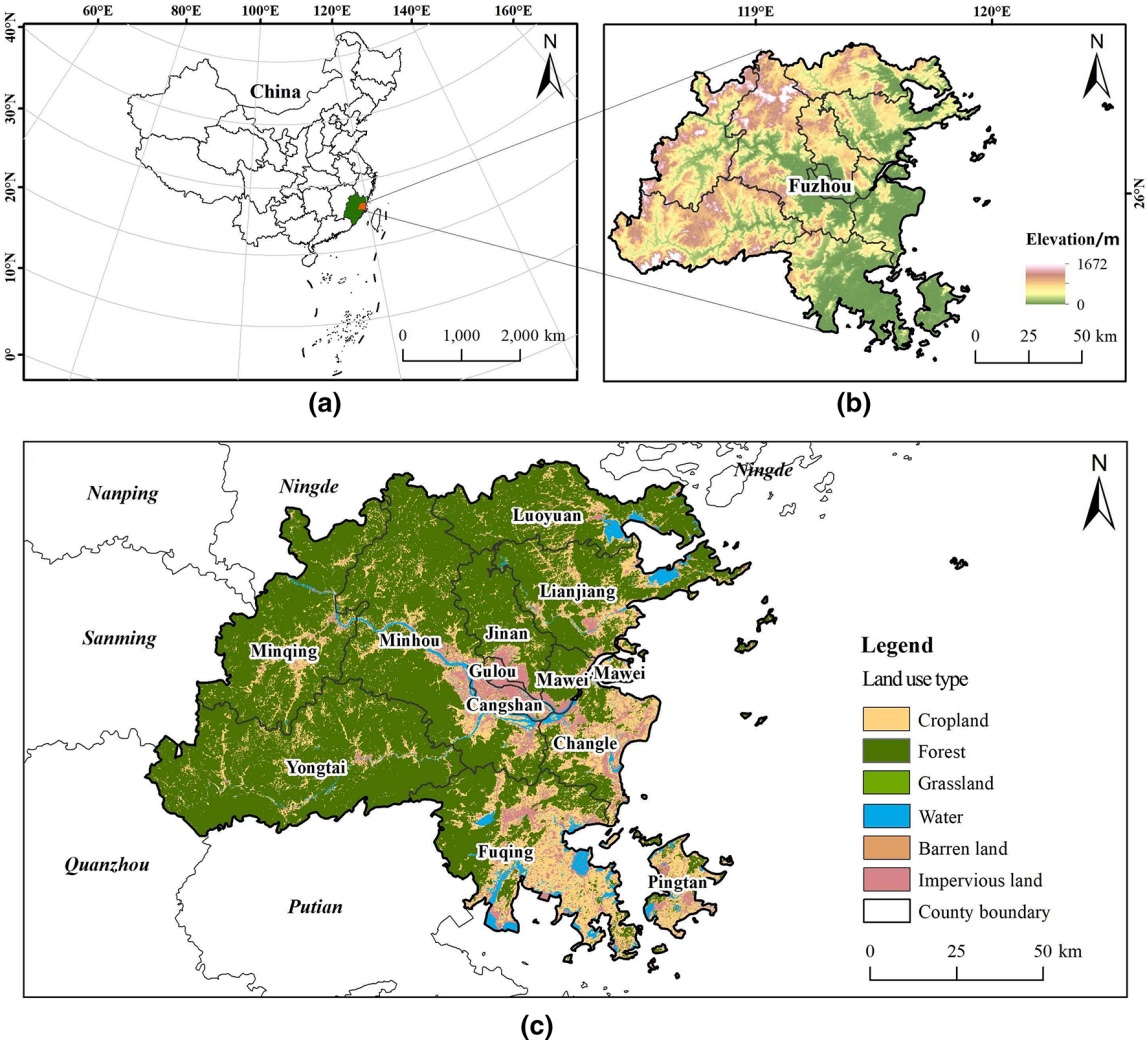
Geographic locations of the study area. (**a**) Fuzhou City in Fujian Province, China. (**b**) Digital elevation model (DEM) image of Fuzhou. (**c**) LULC in Fuzhou. (created by ArcMap, version 10.5, http://www.esri.com/)

Fuzhou City, situated in the southeastern coastal region of China at the confluence of the Min River in eastern Fujian Province (118°6’37” to 119°27’16"E, 25°15’44” to 26°6’34"N), functions as the political, economic, and cultural center of Fujian Province (Fig. 1). The city oversees the administration of six counties and one county-level city, encompassing a total area of 11,968.53 km². The topography of Fuzhou is marked by a high western region and a low eastern region, with an average elevation of 84 m. The landscape is predominantly hilly (40.27%), followed by mountainous (32.41%) and plain areas (27.32%). Fuzhou experiences a subtropical monsoon climate, characterized by an average annual temperature of 21.2 °C. The city boasts an extensive and irregular coastline measuring 800.3 km, which includes a vast maritime area and numerous islands. Key water systems in Fuzhou comprise the Min River, Ao River, Dazhangxi River, and Longjiang River, which predominantly flow from east to west throughout the city.

As one of China’s national central cities, Fuzhou exhibits considerable economic strength, strategic location advantages, and substantial development potential. The city is characterized by diverse topography, abundant mountainous regions, high forest coverage, and a favorable ecological environment. Furthermore, Fuzhou serves as a cultural epicenter, being a significant origin of Min Yue culture, ancient architecture, and maritime culture, and possesses a rich historical and cultural legacy. As of 2023, Fuzhou’s permanent population stands at 8.469 million, with an urbanization rate of 73.91% and a regional GDP of 1.292847 trillion yuan. The city attracted 112.6263 million domestic and international tourists, generating total tourism revenue of 98.389 billion yuan, indicative of a robust recreational market. However, in light of evolving land use patterns and escalating recreational demands, Fuzhou’s ecological spaces are increasingly becoming fragmented^42^. According to the Fuzhou City Statistical Yearbook, in 2023, the forest area in Fuzhou was recorded at 7,307 km², and the wetland area at 563 km², reflecting decreases of 0.11% and 0.35%, respectively, compared to 2022. Additionally, the urban built-up area of Fuzhou expanded from 290.82 km² in 2017 to 410.02 km² in 2022. In this context, Fuzhou faces a significant challenge in reconciling rapid urban development with ecological preservation.

### Data collection and process

The data employed in this research includes three distinct categories: environmental data, Point of Interest (POI) data, and online travel diary data.

The environmental data includes land use data for Fuzhou City in 2023, which was acquired from the Resource and Environmental Science Data Center of the Chinese Academy of Sciences (http://www.resdc.cn). Additional data, such as administrative boundary vector data, Digital Elevation Model (DEM) data, and Normalized Difference Vegetation Index (NDVI) data for Fuzhou City, were sourced from the National Earth System Science Data Center (https://www.geodata.cn). Slope data was generated from the DEM of Fuzhou City and processed using ArcGIS 10.5. Road data was obtained from OpenStreetMap (http://www.openstreetmap.org), while river system data was collected via the Shuijingzhu map downloader (http://www.rivermap.cn).

For the POI data, this study utilizes the Amap Map Open Platform and the “POI Data Query” function provided by “XOmap” (http://www.GIS9.com) to extract a total of 401,452 POI records for Fuzhou City in 2023. Following a rigorous data screening and cleaning process, the final dataset comprised 6,487 valid POI records related to infrastructure and 5,928 valid POI records pertaining to recreational resources. Each record includes pertinent information such as the associated region, name, address, and geographical coordinates.

The online travel diary data encompasses both digital footprint data and web-based textual data. The outdoor travel application “foooooot” (www.foooooot.com), which is widely utilized in China, offers users a platform to document and share their outdoor recreational experiences^43^. Utilizing the Octoparse data extraction tool, detailed information, including photographs and geographic locations shared by users in Fuzhou City from 2014 to 2023, was collected, resulting in a total of 1044 travelogues and 95,227 digital footprint points with geographic coordinates. Additionally, online text data was gathered by crawling travelogues themed focused on “Fuzhou City” from two prominent travel websites, Ctrip (http://www.ctrip.com) and Mafengwo (http://www.mafengwo.cn), also employing the Octoparse data extractor. These platforms are extensively used by Chinese tourists^33^. A total of 1573 travelogues were collected from May 2019 to December 2023, comprising 573 from Ctrip and 1000 from Mafengwo. After conducting manual verification to excluded entries lacking location information or with ambiguous travel routes, 1510 valid travelogues were retained (542 from Ctrip and 968 from Mafengwo). An analysis of these valid travelogues identified 861 geographic locations as recreational flow nodes. The coordinates of each recreational flow node were extracted using the Baidu Coordinate Picker System, and the recreational routes and along with the visitation frequency of each recreational flow node were compiled into a binary matrix for subsequent analysis.

### Research framework and methods

**Fig. 2:**
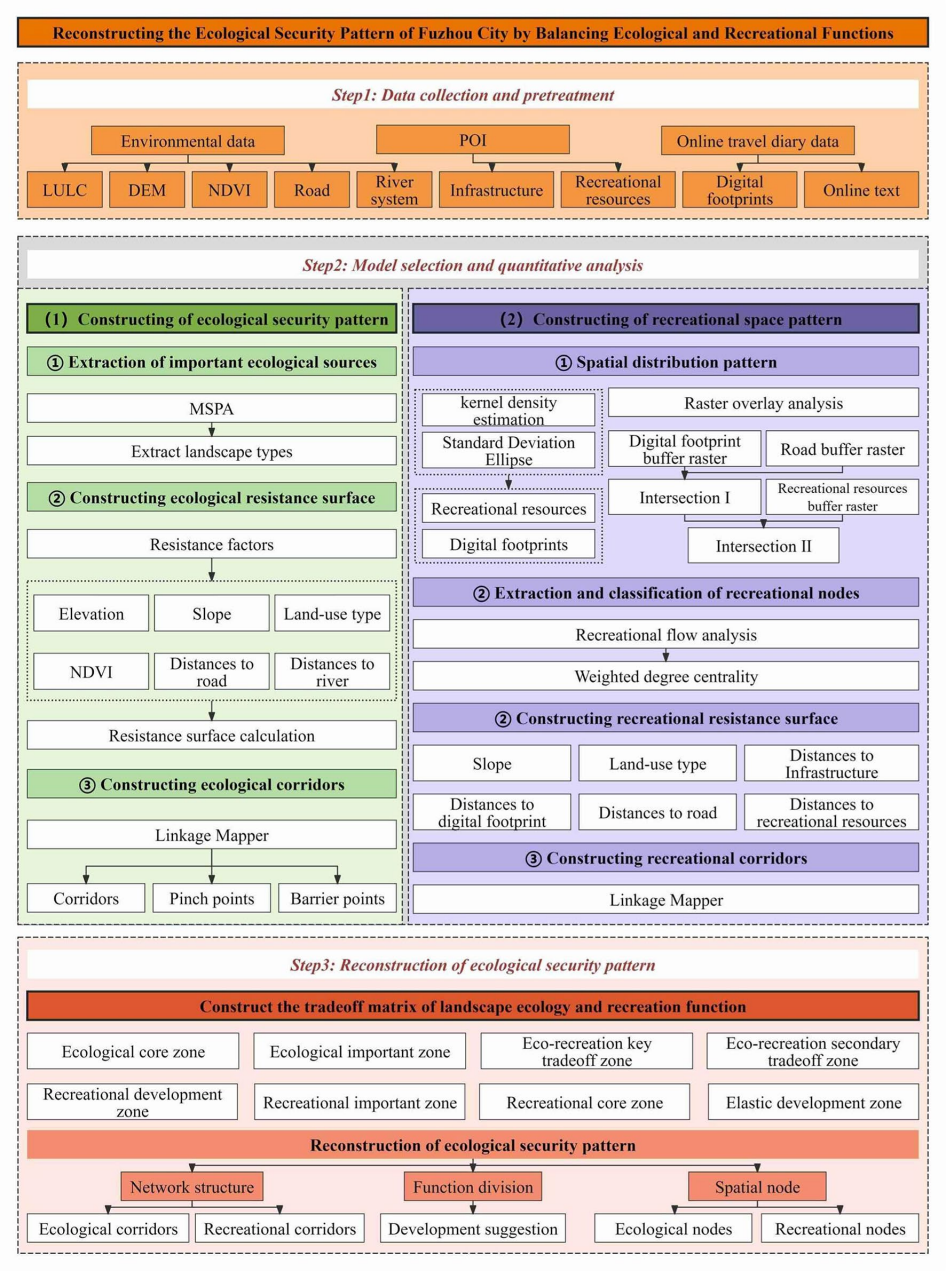
Technical route of the research.

This research develops an ESP that harmonizes urban ecological conservation and recreational development by integrating the ecological and recreational functions of Fuzhou City. Initially, the MSPA model and landscape connectivity assessment are employed to identify the ecological source areas within Fuzhou City. Six resistance factors—namely elevation, slope, land use type, distance to river and roads, and vegetation—are selected to construct the ERS. Subsequently, the Linkage Mapper tool is utilized to extract ECs, ENs, EBPs and EPPs. Following this, a combination of kernel density analysis, raster overlay analysis, standard deviation ellipse analysis, and SNA is applied to identify recreational nodes. Six resistance factors—slope, land use type, distance to roads, infrastructure, digital footprints, and recreational resources—are employed to develop the RRS, with the Linkage Mapper tool again used to extract RCs.Ultimately, utilizing a trade-off matrix method, the relative significance of ecological and recreational functions within each unit is assessed to delineate functional zones and pinpoint strategic points. This process culminates in the reconstruction of the ESP for Fuzhou City, accompanied by recommendations for planning, development, and management strategies. Figure 2 provides a visual representation of the methods and procedures involved in the construction and optimization of the ESP in Fuzhou City, with a comprehensive explanation of each step detailed in the subsequent sections.

#### Construction of ecological security pattern

##### Identification of ESAs and ESPs

In this research, the MSPA model, along with landscape connectivity analysis, was employed to systematically identify and delineate ESAs within the designated study area. Due to the significant vegetation cover and well-established river systems in the study area, forests, grasslands, and water bodies were classified as foreground data, while other land use categories were categorized as background data. The analysis utilized the Guidos Toolbox software, configured with Foreground Connectivity set to 8, Edge Width set to 4, and both “Transition” and “Intext” options activated. A binary raster image of Fuzhou City for the year 2023 was subsequently analyzed. Following the importing of the results into ArcGIS 10.5, seven distinct landscape types were identified: Core Area, Edge Area, Perforation, Bridge Area, Branch, Loop Area, and Islet. Potential core patches with larger surface areas were extracted as preliminary data for the selection of ESAs, based on the landscape connectivity index. The landscape connectivity index of these potential core patches were computed using Conefor software. The dPC value serves as an indicator of the degree to which a particular landscape type facilitates or obstructs the diffusion of ecological flows. Patches were prioritized according to their dPC values, with those exhibiting a dPC value exceeding 0.2 being designated as ecological sources^44^. The geometric centers of the ecological sources were calculated using ArcGIS 10.5, establishing them as ecological source points (ESPs).

Landscape connectivity is defined as the degree of ease or difficulty with which species can traverse between patches, thereby indicating the extent to which a region’s landscape supports species movement. Enhanced landscape connectivity is associated with increased ecosystem stability and spatial continuity^45^. The following equations were utilized in the analysis:

1 $$PC = \frac{{\sum\limits_{{i = 1}}^{n} {\sum\limits_{{j = 1}}^{n} {a_{i} a_{j} p_{{ij}}^{*} } } }}{{A_{l}^{2} }}$$

2 $$dPC = \frac{{PC - PC_{{remove}} }}{{PC}}$$

Where *n* represents the total number of patches; *a*_*i*_ and *a*_*j*_ are the areas of patches *i* and *j*, respectively; *A*_*l*_ represents the cumulative area of the landscape elements; *l*_*ij*_ indicates the shortest distance between patches *i* and *j* ; p^*^_ij_ reflects the maximum probability of species dispersal between patches *i* and *j*; *PC*_*remove*_ refers to the overall index following the removal of a patch. *PC* indicates the probability of connectivity, and *dPC* evaluates the significance of each patch in sustaining connectivity by quantifying the alterations in *PC*.

##### Construction of ERS

The development of a comprehensive ERSthrough the systematic screening and assessment of resistance factors enables a more accurate measurement of the impediments to ecological components during their movement, thereby aiding in the identification of potential ECs. In this research, resistance factors were selected based on prior studies and the specific environmental conditions of Fuzhou City, which included variables such as elevation, slope, land use type, distance to river and roads, and NDVI. The resistance values for each factor in this study are determined with reference to the values used in previous studies^15,44,46,47^ (Table 1). Factors such as elevation, slope, NDVI, and land use type significantly affect the spatial distribution of species activities and resources availability. The distance to river system enhances biological activity, whereas roads serve as physical barriers to species migration, complicating movement for species located near these infrastructures. The weights assigned to each factor were established using the Analytical Hierarchy Process (AHP) within yaahp software, followed by a consistency assessment. The results for each factor were integrated with their respective weights to formulate a comprehensive ERS for Fuzhou City. The AHP is a multi-criteria, multi-objective decision-making method that combines qualitative and quantitative approaches, making it particularly effective for addressing complex problems that resist full quantification. This method decomposes complex decision problems into hierarchical structures, such as goals, criteria, and alternatives, and subsequently evaluates the relative significance of each indicator based on the experience and expertise of specialists in the field.

**Table 1:** Ecological resistance factor index system and weights.

Restraint factor	Resistance value					Weight
	1	2	3	4	5	
Elevation/m	< 20	50–100	100–200	200–500	> 500	0.21
Slope/°	< 5	5–10	10–15	15–20	> 20	0.11
Land use type	Forestland	Grassland	Water area	Farmland	Other land	0.28
Distances to river/km	< 100	50–100	100–150	150–200	> 200	0.14
Distances to road/km	> 1000	500–1000	200–500	100–200	< 100	0.09
NDVI	> 0.5	0.3–0.5	0.1–0.3	0–0.1	< 0	0.17

##### Construction of ECs

The Linkage Mapper toolbox, an essential analytical instrument in circuit theory, was employed to simulate the stochastic dynamics of species migration and identify the least-cost pathways connecting ecological sources^47^. Utilizing the Linkage Pathways tool within Linkage Mapper 2.0 and employing the ERS as foundational data, the least-cost paths for ecological and energy flows were calculated to elucidate the spatial distribution of ECs. The Centrality Mapper module facilitated the quantification of the significance of various corridors by identifying their centrality, subsequently categorizing these corridors into primary and secondary classifications through the application of the natural breaks method.

EPPs are defined as regions within ecological corridors that exhibit elevated current density. These regions are marked by concentrated voltage due to high resistance in the surrounding areas, resulting in robust currents that provide substantial ecological protection value^6^. The Pinchpoint Mapper tool within Linkage Mapper was utilized to identify these pinch points, employing the “All to one” mode. Following the establishment of corridor cost-weighted distance at 1000 m, 1500 m and 2000 m, it is empirical testing revealed that when the corridor cost-weighted distance exceeds 1500 m, the core positioning of the EPPs remains unaffected by variations in corridor width. Consequently, this study adopted a corridor cost-weighted distance of 1500 m. The cumulative current map was generated by aggregating the current across all Ecological Source Areas (ESAs), and the centrality values of each ESPs and ECs were calculated to identify the locations of EPPs.

EBPs are identified as areas that obstruct species migration and dispersal between ESPs. The restoration of these points is anticipated to enhance landscape integrity and connectivity while mitigating migration resistance. The Barrier Mapper tool in Linkage Mapper was utilized in “Maximum” mode, with a minimum search radius of 100 m and a maximum of 300 m, employing a step size of 100 m. A moving window approach was implemented to identify barrier points, wherein the central pixel values within the search window were substituted with the least-cost distance values between source patches. The enhancement in connectivity post-barrier removal was represented by the unit improvement in least-cost distance. Areas exhibiting high values were esignated as EBPs within the ECs.

#### Construction of RSP

##### Analyzing the spatiality of recreational resources and digital footprints

By integrating the spatial distribution of recreational resources and visitor preferences, a more focused ESP framework can be developed. Kernel Density Estimation (KDE) is utilized to examine the spatial density characteristics and distribution patterns within the study area, as well as to evaluate the effects on adjacent regions. KDE is employed to visualize recreational resources and digital footprints^48^, represented by the following formula:

3 $$f\left( x \right) = \frac{1}{{Nh}}\sum\limits_{{i = 1}}^{n} k \left( {\frac{{x - x_{i} }}{h}} \right)$$

Where *f(x)* represents the value of kernel density analysis at position *x*; h signifies the bandwidth; *N* represents the number of points within the bandwidth distance from position *x*_*i*_; And *K* is a spatially weighted kernel function.

The Standard Deviation Ellipse (SDE) is a traditional technique for analyzing directional distribution directions of spatial data, facilitating a quantitative assessment of the centrality, distribution, spatial configuration, and other directional attributes of recreational resources and digital footprints data within the study area^15^. This method is applied to investigate the directional changes of recreational resources and visitor digital footprints in Fuzhou City from 2014 to 2023.

Prior studies suggest that the grid overlay analysis method, which incorporates roads, digital footprints, and recreational resources, provides a theoretical foundation and practical framework for the scientific identification and assessment of recreational potential areas, as well as for the constructing RCs^49^. ArcGIS 10.5 is employed to create buffers around the roads, digital footprints, and recreational resources data in Fuzhou City. Following raster processing, the raster calculator is utilized for overlay analysis, and the resultant raster data serves as the basis for selecting RNs in Fuzhou City.

##### Recreational flow analysis and RNs selection

In this research, SNA was utilized to develop a network representing recreational flow. Data matrices obtained from online travel diaries were analyzed using Gephi software, resulting in the establishment of a recreational flow network for Fuzhou City. RNs were identified and categorized based on weighted degree centrality, a metric that assesses the positioning and significance of nodes within the recreational flow network by indicating the degree of connectivity among them. This metric encompasses both inflow and outflow degrees. A higher centrality value for a node signifies its crucial role within the network, reflecting its greater attractiveness and influence in the recreational market. In studies of recreational flow network, this index proves advantages in pinpointing key nodes and their essential interconnections. Prior Research has indicates that nodes exhibiting elevated weighted degree centrality tend to fulfill more prominent functional roles and possess greater importance within the network^33^. The formula for calculating this index is as follows:

4 $$WD_{i} = \sum\nolimits_{j}^{N} {w_{{ij}} } + \sum\nolimits_{j}^{N} {w_{{ji}} }$$

where *WD*_*i*_ represents weighted degree centrality, *DI*_*i*_ is the diffusion index, *w*_*ij*_ represent the weights of the edges between nodes *i* and *j* in both directions, and *w*_*ji*_ represents the weights of the edges between nodes *j* and *i* in both directions.

In this study, nodes with weighted degree centrality values exceeding the average were classified as strong functional nodes and identified as potential recreational nodes, whereas those with values below the average were categorized as weak functional nodes^50^. Taking into account the specific characteristics of Fuzhou City, a thorough evaluation incorporating KDE, SED, grid overlay analysis, and the identification of potential RNs was conducted. Nodes exhibiting high weighted degree centrality, situated in areas characterized by elevated values in KDE and grid overlay analysis, were prioritized as RNs.

##### Construction of RRS and RCs

The construction of the RRS necessitated an examination of both natural geographical factors elements and socioeconomic factors, as well as insights into public preferences and behavioral patterns. This research, informed by prior studies^27,47,51^ and the unique context of Fuzhou City, identified slope, land use type, distance to roads, infrastructure, digital footprints, and recreational resources as resistance factors, to which resistance coefficients were assigned accordingly. Slope and land use type significantly affect the spatial extent, comfort, and safety associated with recreational activities. Roads, as vital connections between various recreational areas, directly influence the spatial distribution and flow dynamics of the recreational network. Infrastructure plays a crucial role in facilitating recreational activities and ensuring their effective operation. The digital footprint serves as an indicator of recreational preferences and the intensity of engagement in recreational activities, while recreational resource constitute the foundation elements for the development of the recreational network and are pivotal in attracting tourists. The AHP was employed using the yaahp software to ascertain the weight of each factor (Table 2), followed by a consistency assessment. The results for each factor were subsequently integrated with their weights to formulate a comprehensive RRS for Fuzhou City. Using the Linkage Pathways tool within the Linkage Mapper 2.0 toolbox, and employing the RRS as input data, the least-cost paths for recreational flow were calculated to delineate the spatial distribution of recreational corridors. By quantifying the centrality of corridor flow, the corridors were further categorized based on their significance. In this study, the cost-weighted distance threshold for truncating recreational corridors was established at 1500 m.

**Table 2:** Recreational resistance factor index system and weights.

Restraint factor	Resistance Value					Weight
	1	2	3	4	5	
Slope/°	< 5	5–10	10–15	15–20	> 20	0.15
Land-use type	Forestland	Grassland	Water area	Farmland	Other land	0.13
Distances to infrastructure/km	< 500	500–1000	1000–1500	1500–2000	> 2000	0.09
Distances to digital footprint/km	< 500	500–1000	1000–1500	1500–2000	> 2000	0.19
Distances to road/km	< 100	100–200	200–500	500–1000	> 1000	0.24
Distances to recreational resource/km	< 500	500–1000	1000–1500	1500–2000	> 2000	0.21

#### Reconstruction of ecological security pattern

**Fig. 3:**
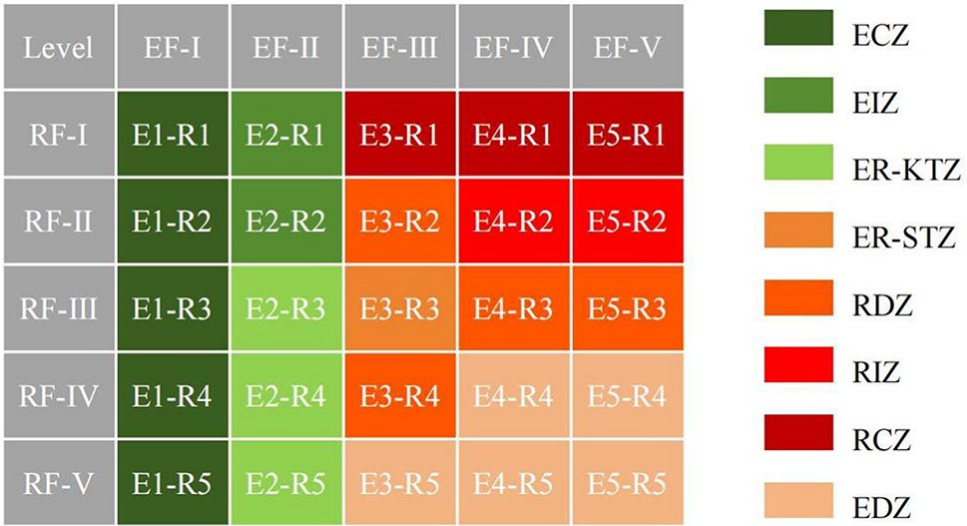
Eco-recreation function tradeoff matrix.

This study develops a tradeoff matrix to evaluate the relative significance of ecological and recreational functions across various units in Fuzhou City, based on their ecological and recreational function levels^52^. Utilizing the findings from the ERS and RRS analyses, the ecological and recreational functions of Fuzhou City were categorized into five levels, ranging from high to low (I, II, III, V), resulting in a total of 25 combinatorial outcomes (Fig. 3). The tradeoff matrix facilitates a comparative analysis of the ecological and recreational functions across different regions, leading to the classification of these outcomes into eight functional zones.

The Ecological Core Zone (ECZ) is identified as the central areas of the ecosystem, characterized by exceptionally high ecological value, sensitivity, and vulnerability. These regions typically encompass unique natural ecosystems, exhibit rich biodiversity, and provide essential ecological services, while also being particularly sensitive to anthropogenic impacts. Conversely, the Ecological Important Zone (EIZ) functions as a transitional buffer surrounding the core zone, primarily aimed at safeguarding the integrity and stability of ESAs. Although the ecological functions and biodiversity in these areas may be lower than those in core zones, they still possess considerable ecological significance.

The Recreational Core Zone (RCZ) is identified as the area with the highest concentration of recreational activities, supported by well-developed infrastructure. This zone is generally situated at the center of recreational spaces and serves as the primary venue for both residents and tourists. The Recreational Important Zone (RIZ) and the Recreational Development Zone (RDZ) are designed to complement and extend the functions of the core zone. Specifically, the RIZ, which encircles the core zone, possesses high recreational value and plays a vital strategic role in enhancing the regional recreational appeal for recreation, alleviating pressure on the core zone, and promoting a balanced spatial distribution of recreational activities. In contrast,,the RDZ is conducive to the development of outdoor recreational activities and the sustainable utilization of both natural and cultural resources.

The Eco-recreation Key Trade-off Zone (ER-KTZ) necessitates meticulous management to achieve a balance between ecological preservation and recreational activities. Although these areas exhibit high ecological value, their potential for recreational development is relatively constrained, necessitating rigorous planning and management to ensure a harmonious coexistence. In comparison, the Eco-recreation Secondary Trade-off Zone (ER-STZ) possesses moderate ecological value and recreational development potential, rendering the tradeoff between the two less critical, yet still requiring attention to their balance and optimization. Furthermore, the Elastic Development Zone (EDZ), characterized by lower ecological value and recreational potential, provides greater flexibility in reconciling ecological protection with recreational development. This zone allows for dynamic adjustments and optimizations based on actual ecological conditions and socioeconomic development needs, thereby exploring diverse pathways for recreational development while ensuring ecological security.

Strategic points are defined as critical locations within the ESP framework. In this study, the intersections of ECs and RCs are designated as strategic points, with intersections of primary corridors classified as major strategic points and intersections of secondary corridors classified as minor strategic points.

## Results

### Construction of ESP

#### Extraction of ESAs and ESPs

**Fig. 4:**
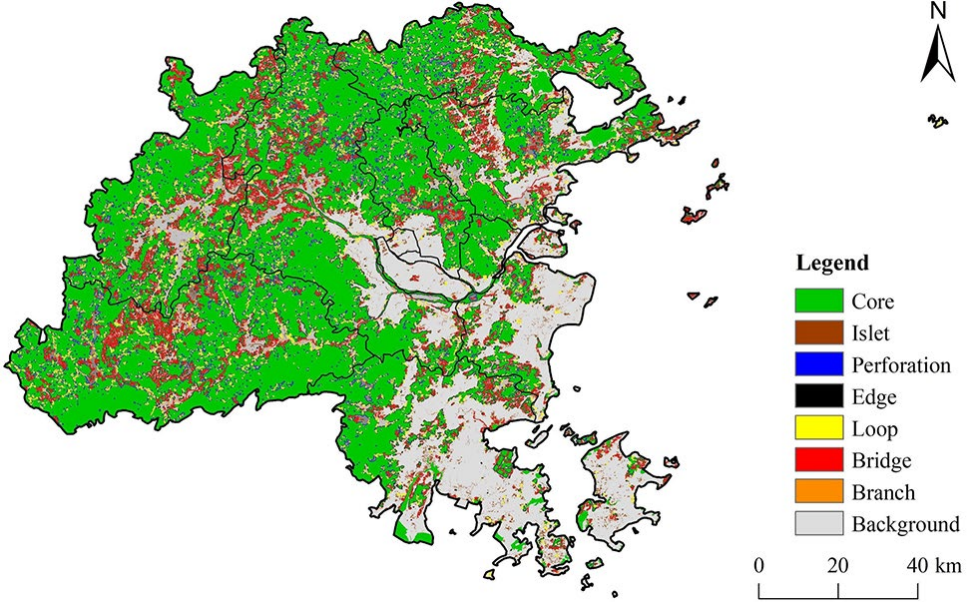
Results of MSPA analysis in Fuzhou City. (created by ArcMap, version 10.5, http://www.esri.com/)

The results of the MSPA analysis results for Fuzhou City are presented in Fig. 4, which indicate the presence of seven different landscape types. The most significant type is the Core Area, which make up 49% of the landscape and is mainly found in the western and northern parts of the study area. While there are several Core Areas in the central and southeastern regions, their smaller and more scattered nature limits the connectivity between ecological source areas.

**Fig. 5:**
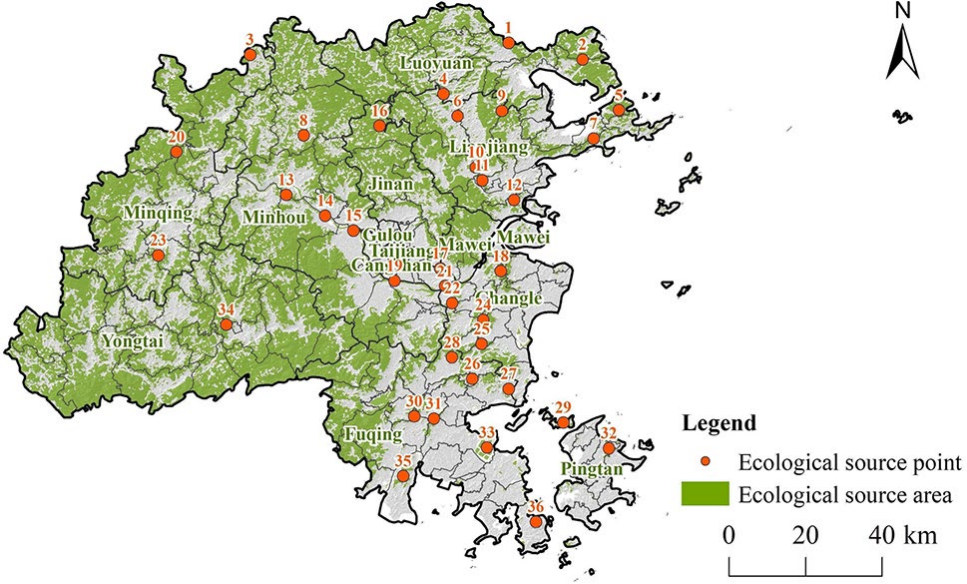
Spatial distribution of ESAs and ESPs. (created by ArcMap, version 10.5, http://www.esri.com/)

The MSPA analysis identified 36 core patches with *dPC* values exceeding than 0.2, which were then ranked by size. From this, 36 habitat patches larger than 1 km² were designated as ESAs for Fuzhou City’s ESP (Fig. 5). These ESAs cover a total area of 5807.90 km², representing 48.53% of Fuzhou City’s overall area. The distribution of these ESAs is uneven: the mountainous and hilly areas in the western and northern sections have fewer but larger ESAs, while the eastern and southern plains contain many smaller and more dispersed ESAs. Intensive land use and ongoing encroachment on ecological land have led to significant landscape fragmentation. Differences in land use demands across various districts have resulted in insufficient connectivity in the ecological systems between the eastern and western regions of the watershed. To address this issue, it is essential to improve connectivity between patches by establishing ECs to reduce landscape fragmentation and enhance ecosystem services in the area.

#### Construction of ERS

**Fig. 6:**
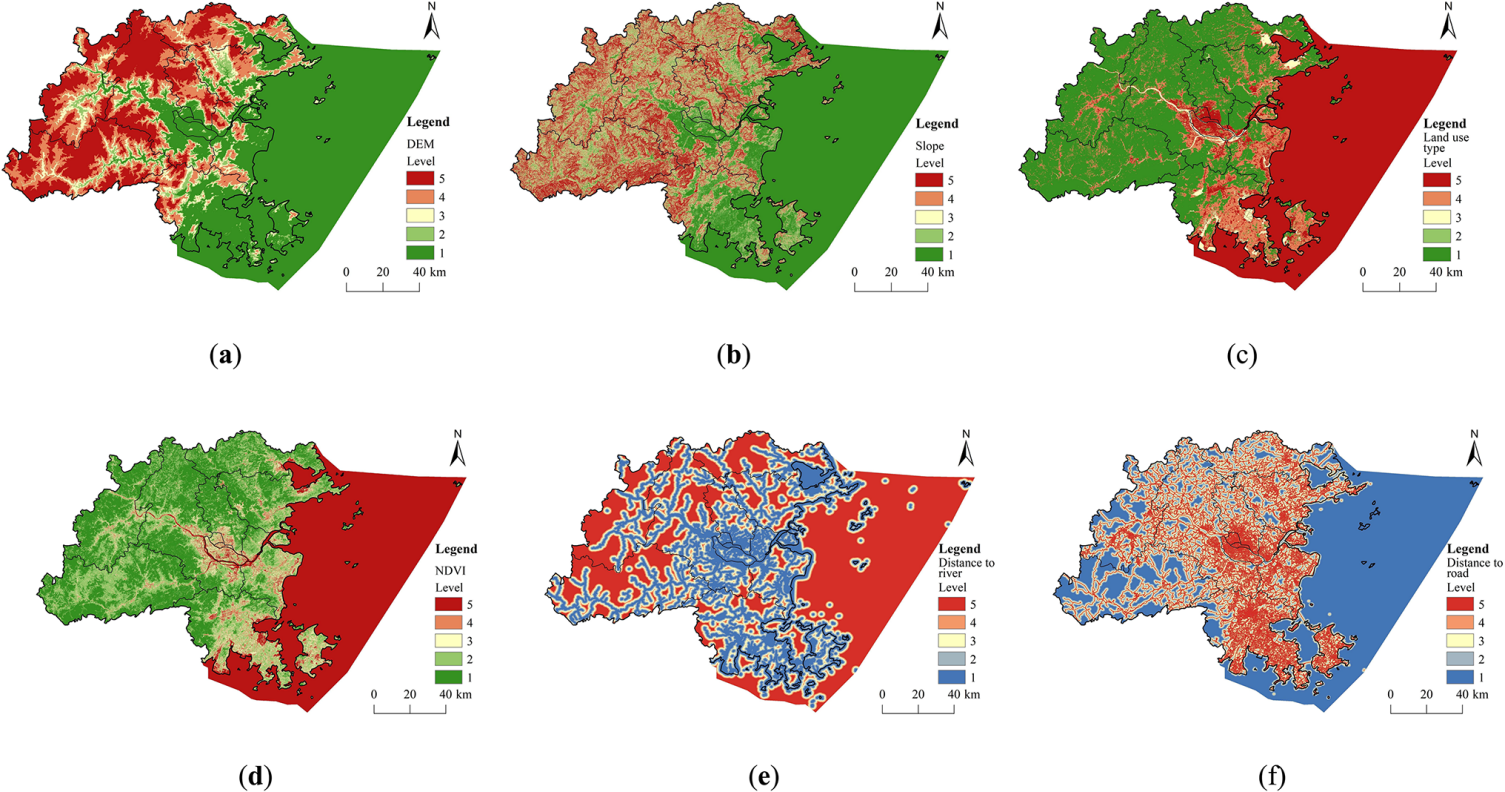
Classification of resistance levels of each ecological resistance factor. (created by ArcMap, version 10.5, http://www.esri.com/) (**a**) DEM; (**b**) Slope; (**c**) Land use type; (**d**) NDVI; (**e**) Distance to river; (**f**) Distance to road.

This research created layers for each resistance factor in ArcGIS (Fig. 6) based on the evaluation criteria and their respective weights.

**Fig. 7:**
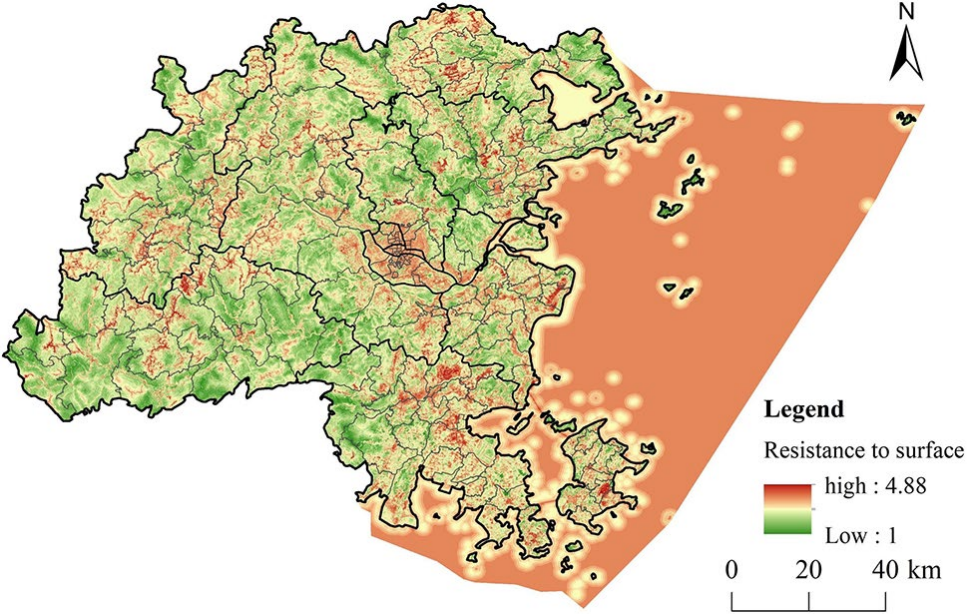
Comprehensive ERS. (created by ArcMap, version 10.5, http://www.esri.com/)

A weighted sum of these resistance factors was calculated using the raster calculator to generate the comprehensive ERS for Fuzhou City (Fig. 7). The resistance values in the area exhibit a trend of high values in the east, low values in the west, and high values surrounded by lower ones in the center, indicating considerable spatial variation. The areas with high resistance are primarily situated in the central urban development zones of Fuzhou City, while low resistance areas are primarily located in the mountainous and river valley regions to the west and north.

#### Extraction of ecological source

**Fig. 8:**
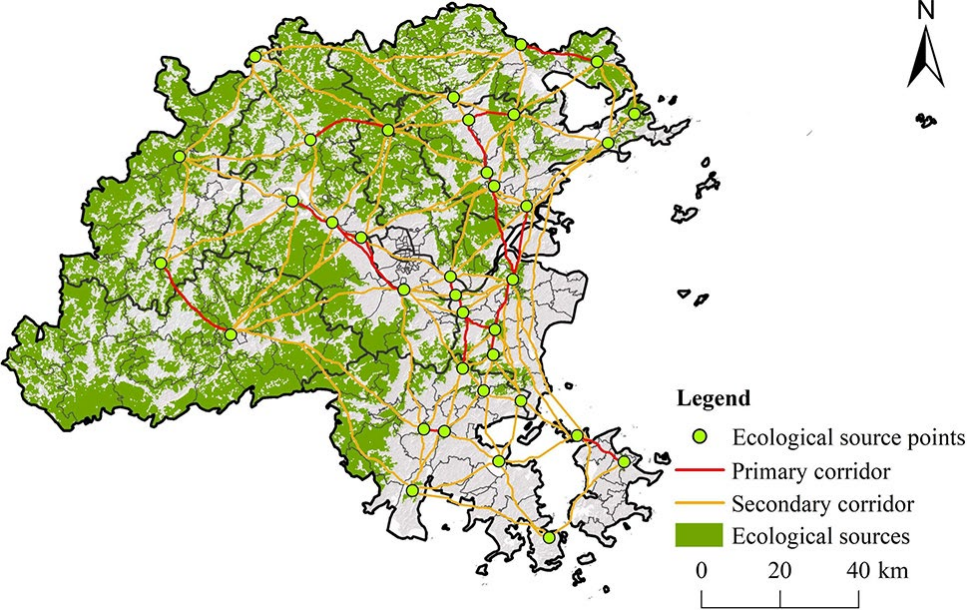
Distribution of Ecological corridors. (created by ArcMap, version 10.5, http://www.esri.com/)

Using circuit theory, a total of 98 ECs were simulated, covering a total length of 2500.55 km (Fig. 8). The Centrality Mapper tool was eutilized to evaluate their significance, and natural breaks classification was applied to the results, leading to the identification of 18 primary corridors and 80 secondary corridors. The spatial distribution of ECs in Fuzhou City reveals a higher density in the east region and a lower density in the west. The ESPs in the central part of the study region are abundant and densely distributed, resulting in shorter and more concentrated ECs.

**Fig. 9:**
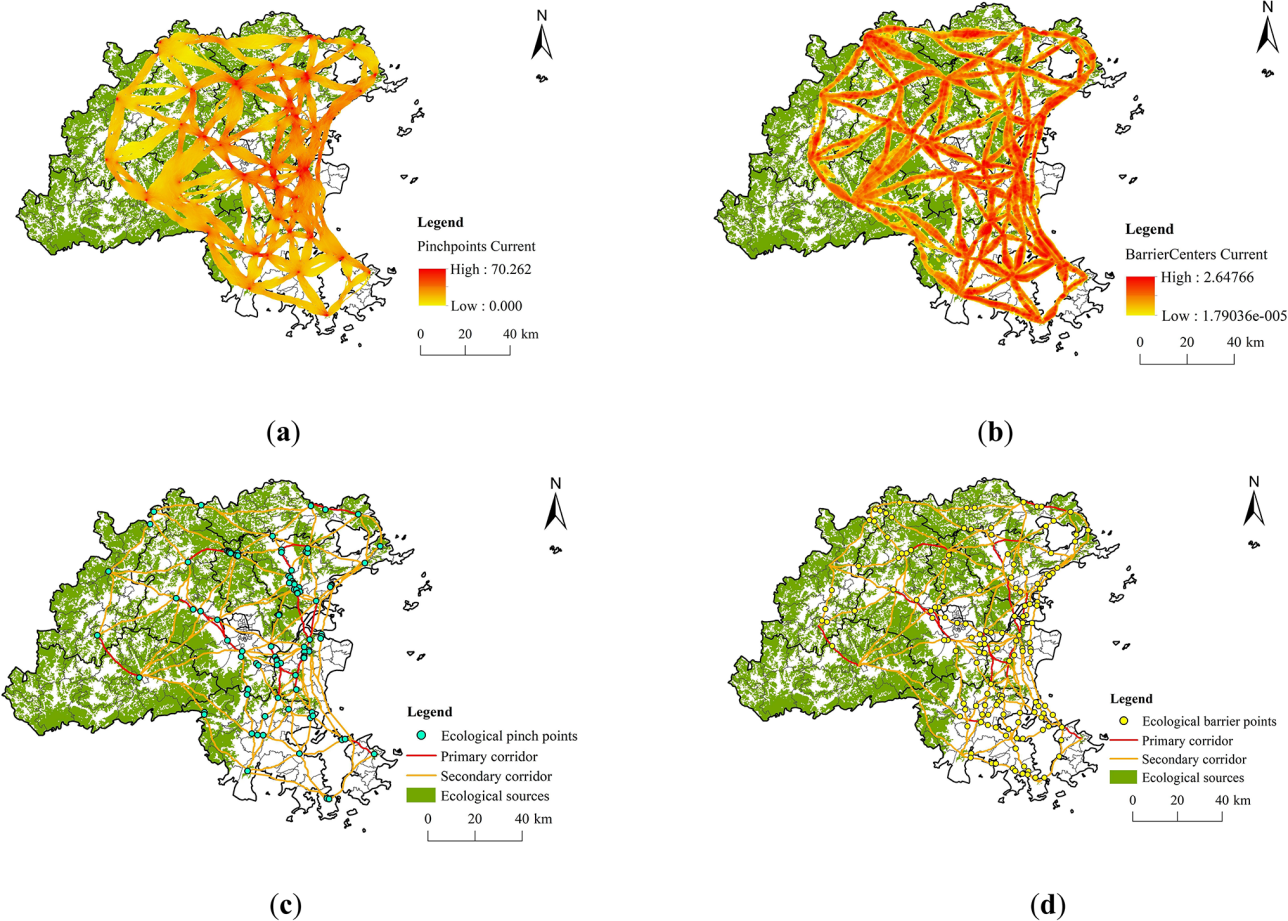
Spatial distribution of EPPs and EBPs. (created by ArcMap, version 10.5, http://www.esri.com/) (**a**) Pinchpoints current; (**b**) Barriercenters current; (**c**) Pinch points; (**d**) Barrier points.

The Pinchpoint Mapper tool pinpointed 100 high-value pinchpoint areas (Fig. 9). In general, EPPs are primarily found in the central and eastern sections of the study area, characterized by land types such as forests, water bodies, and croplands. These regions exhibit lower resistance values and are crucial for maintaining ecological stability and improving landscape connectivity. Additionally, the Barrier Mapper tool identified 146 high-value EBPs, mainly situated in the central and eastern urban areas, where land use is largely focused on development. In these areas, human activities are prevalent, ecological functions are diminished, and landscape connectivity is poor, leading to increased ecological resistance values.

### Construction of RSP

#### Spatial analysis of recreational resources and digital footprints

**Fig. 10:**
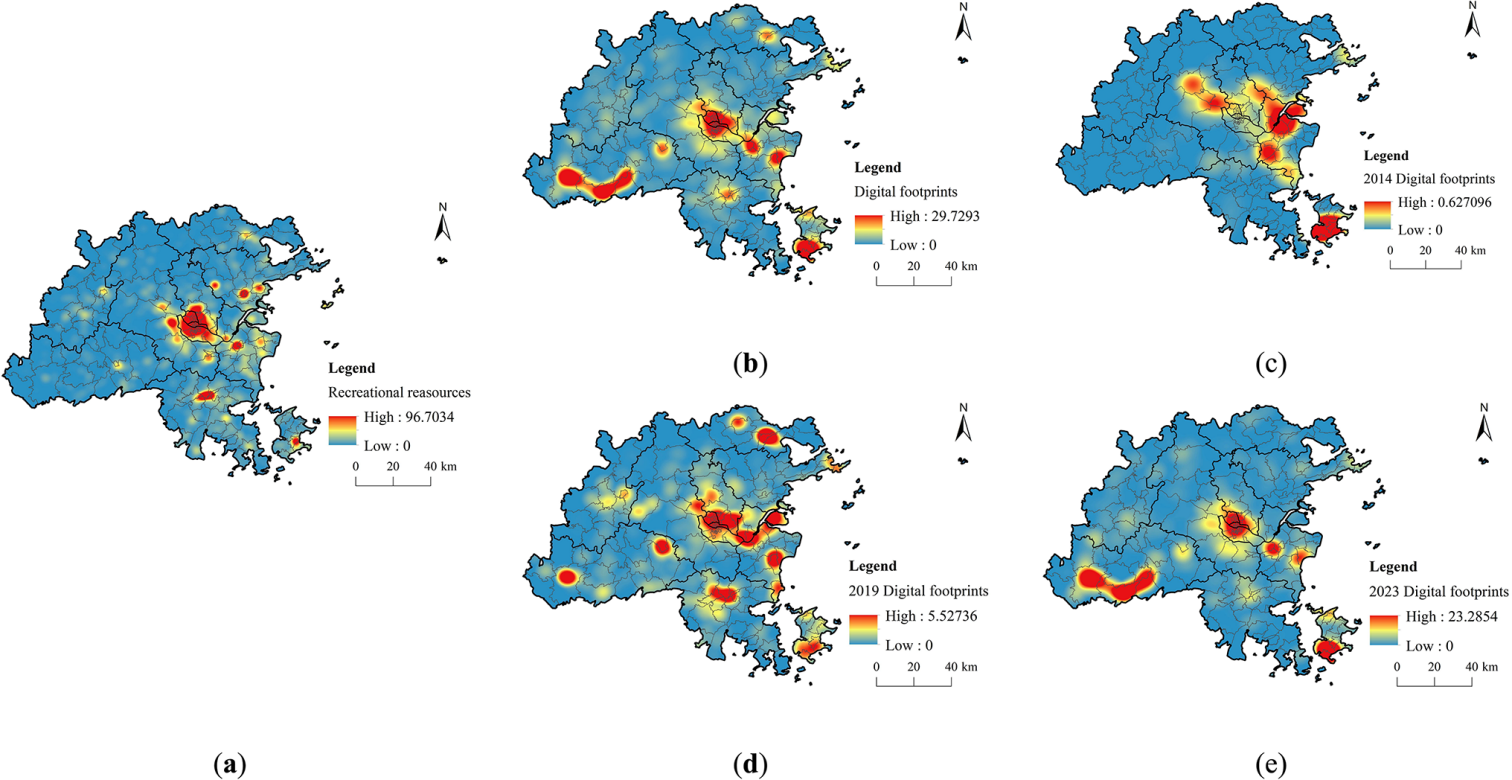
KDE of recreational resources and digital footprints. (created by ArcMap, version 10.5, http://www.esri.com/) (**a**) Recreational resources; (**b**) 2014–2023 digital footprints; (**c**) 2014 digital footprints; (**d**) 2019 digital footprints; (**e**) 2023 digital footprints.

KDE and SDE were utilized to analyze the spatial distribution and development trends of recreational resources and digital footprints in Fuzhou City. The findings reveal a general trend of higher density in the eastern section of the city compared to the west, with a clustered distribution in the east and a more dispersed one in the west (Fig. 10). The spatial distribution of digital footprints indicates a movement from the central urban areas to the peripheral regions, suggesting that the appeal of recreational resources in the western mountainous regions of Fuzhou is on the rise, resulting in an increased demand for recreational activities in ecologically favorable, along with significant inter-regional migration.

**Fig. 11:**
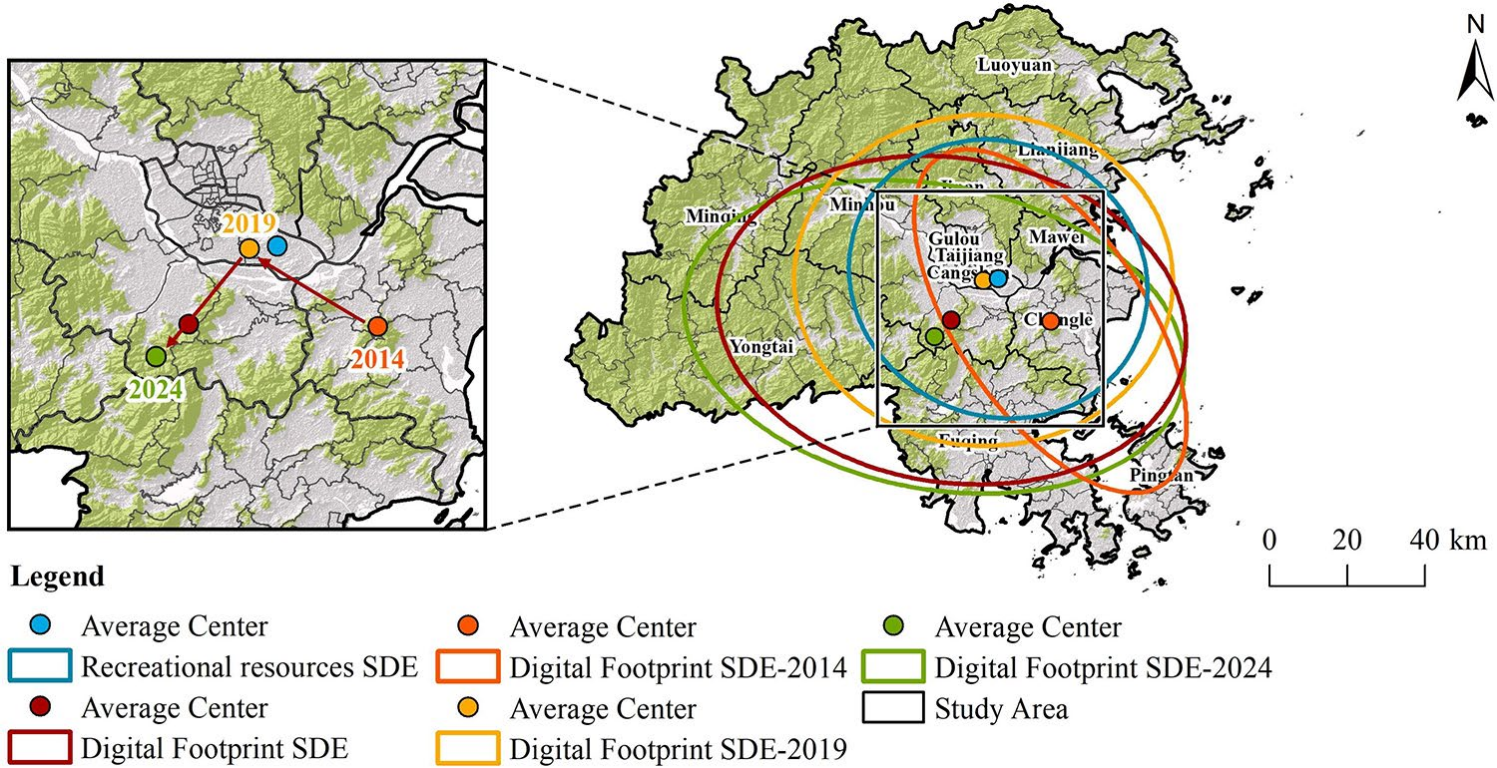
Distribution of Ecological corridors. (created by ArcMap, version 10.5, http://www.esri.com/).

The centers of the standard deviation ellipses for digital footprints and recreational resources are located in Minhou County and Cangshan District, respectively. Over time, the center of the digital footprint’s standard deviation ellipse was located in Changle District in 2014, then shifted 18.86 km northwest to Cangshan District by 2019, and subsequently moved 18.47 km southwest to Minhou County by 2024. The movement of the average center and the orientation of the ellipse in the SDE further support this trend (Fig. 11). Consequently, when planning the recreational network in Fuzhou City, it is crucial to not only link recreational resources but also cater to the recreational needs of residents and visitors in the western regions, thereby improving connectivity between the eastern and western parts of the city.

77

**Fig. 12:**
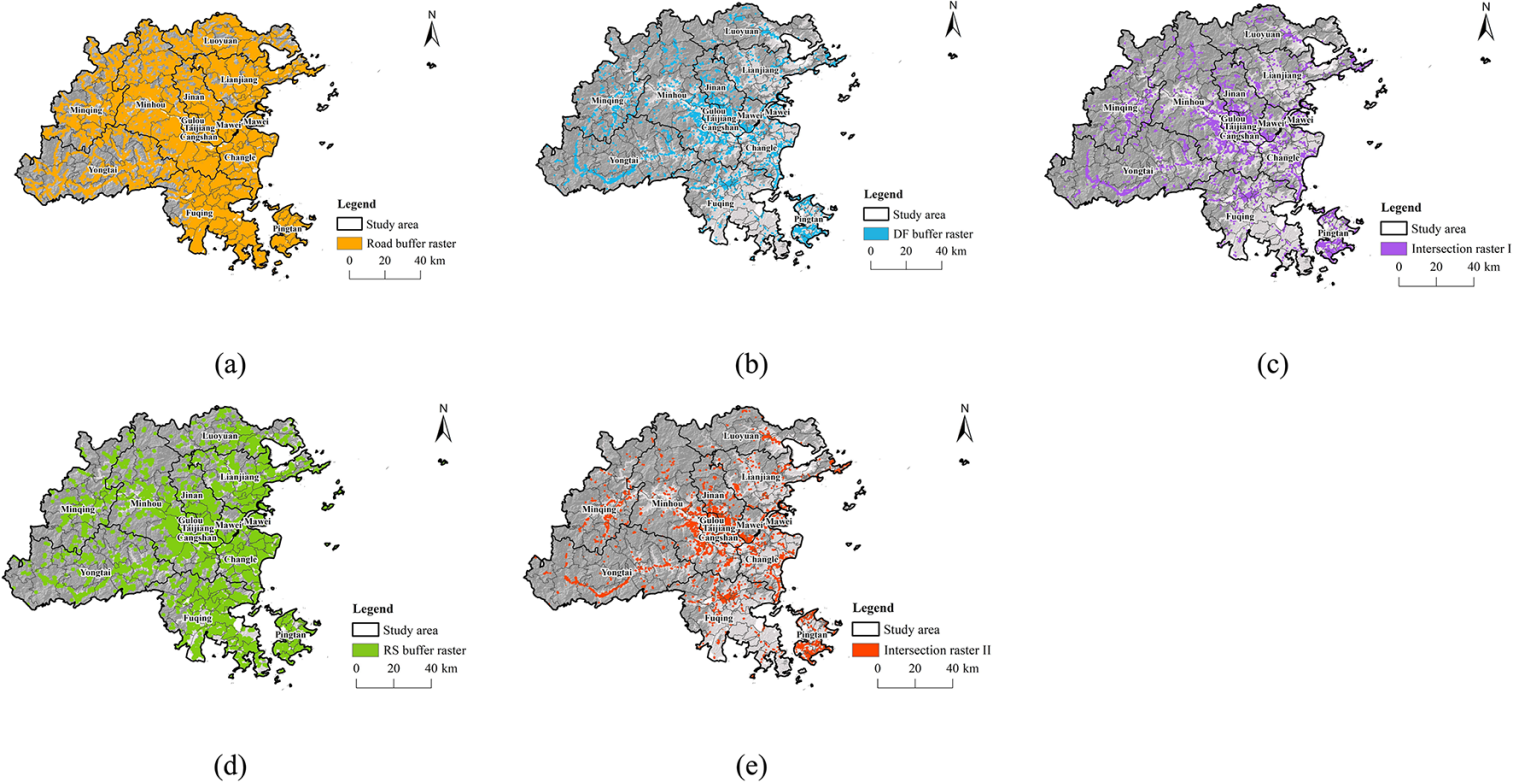
Raster overlay analysis. (created by ArcMap, version 10.5, http://www.esri.com/) (**a**) Road buffer raster; (**b**) Digital footprints buffer raster; (**c**) Intersection raster I; (**d**) Recreational resources buffer raster; (**e**) Intersection raster II.

To accurately pinpoint significant recreational areas in Fuzhou City, a multi-source data overlay analysis was conducted based on relevant studies (Fig. 12). Using ArcGIS, buffers of 0.5 km were established around the roads and digital footprints of Fuzhou City, which were then rasterized. An intersection was derived from these rasters to create Intersection Raster (I) Subsequently, a 1 km buffer was generated and rasterized around the recreational resources in Fuzhou City. The intersection of Raster I with the raster of recreational resources was then extracted to form Intersection Raster (II) The spatial distribution patterns of these regions serve as essential reference points for selecting RNs in Fuzhou City.

#### Recreation flow construction results and RNs selection

**Fig. 13:**
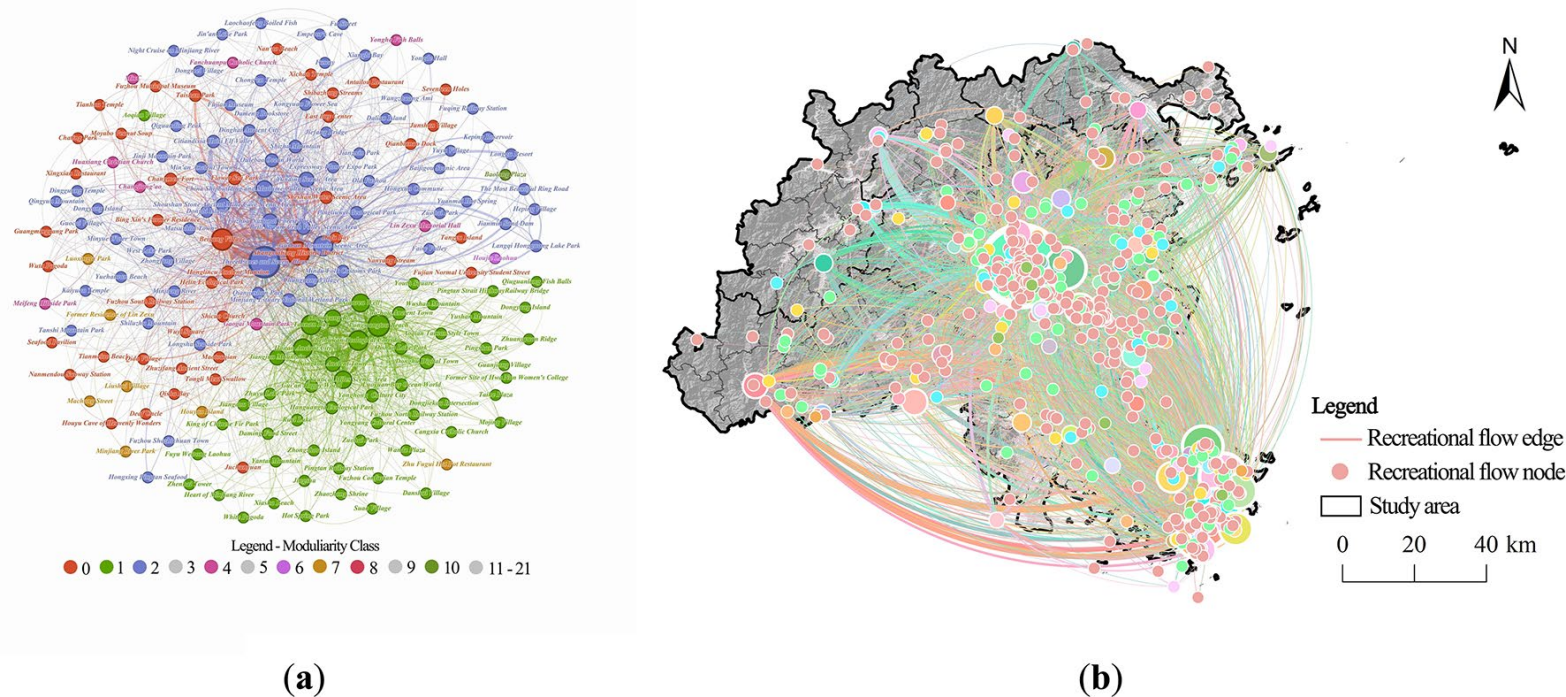
Analysis of recreational flow network. (**a**) Weighted network map (top 21%). (created by Gephi, version 0.10.1, https://gephi.org/) (**b**) Spatial relation characteristics. (created by ArcMap, version 10.5, http://www.esri.com/).

Using Gephi 0.10.0, a recreational flow network was constructed with node weights determined by weighted degree centrality, resulting in a network distribution map for Fuzhou City’s recreational flow weighted degree centrality and a spatial connectivity feature map of recreational flows (Fig. 13). The analysis in Gephi revealed an average weighted degree centrality of 8.545. Out of the 184 recreational flow nodes, 21% had a weighted degree centrality exceeding the average, categorizing them as strong functional nodes.

**Fig. 14:**
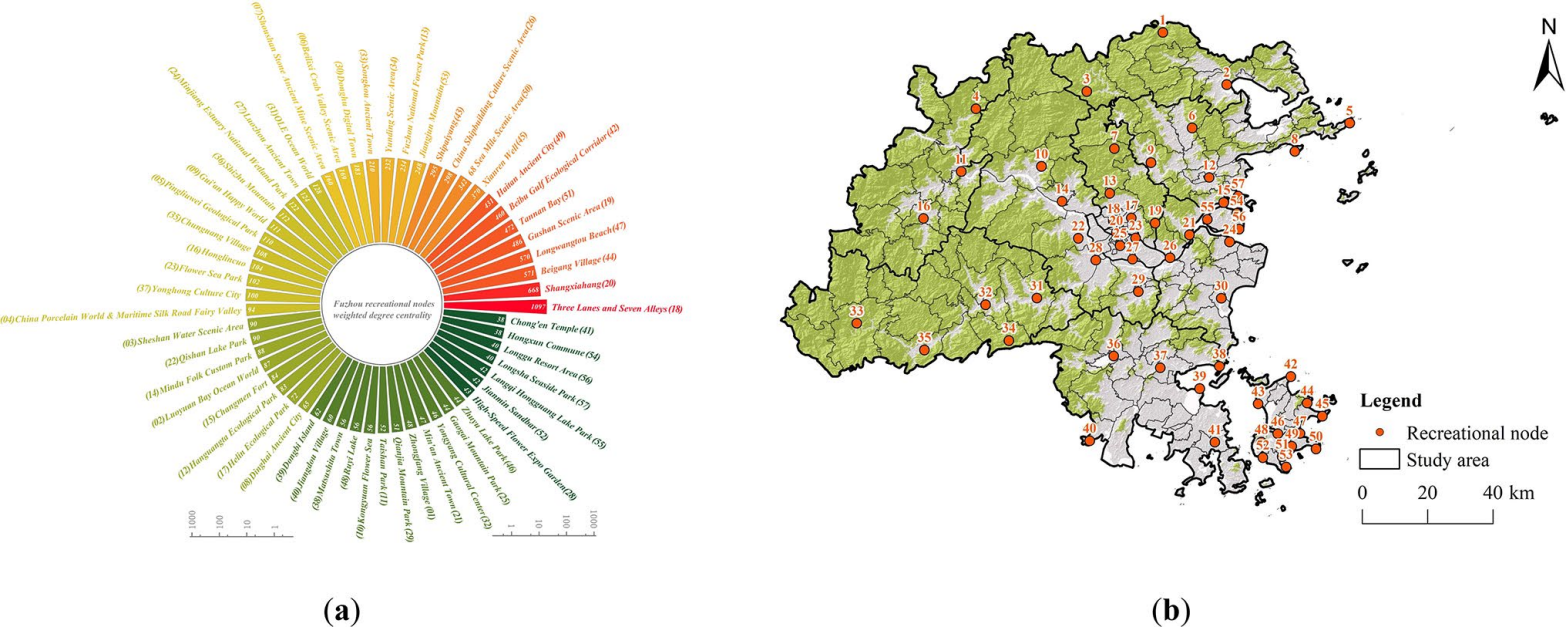
Analysis of RNs. (**a**) Weighted degree centrality of RNs; (created by Gephi, version 0.10.1, https://gephi.org/) (**b**) Spatial distribution of RNs. (created by ArcMap, version 10.5, http://www.esri.com/).

Based on previous KDE, SDE, and raster overlay analyses, and integrating the results from the recreational flow analysis with the characteristics of nearby recreational resources, a total of 57 RNs were identified (Fig. 14). These nodes were visualized in ArcGIS to enhance the understanding of their spatial distribution. The RNs in Fuzhou City are primarily located in the central urban areas, the eastern coastal regions, and Pingtan Island, showing a spatial distribution that is denser in the east and more sparse in the west.

#### Construction of RCs

**Fig. 15:**
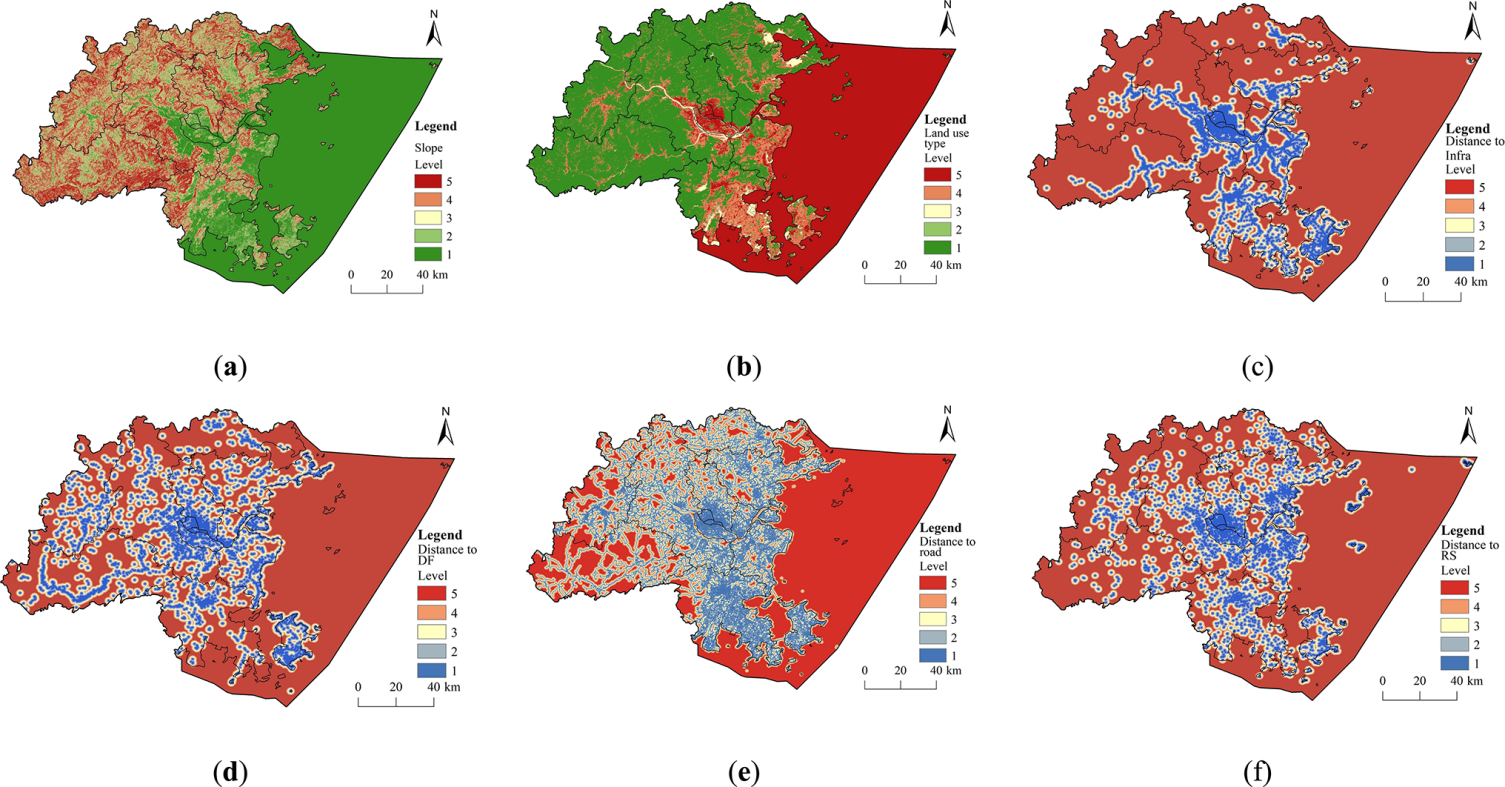
Classification of resistance levels of each recreational resistance factor. (created by ArcMap, version 10.5, http://www.esri.com/) (**a**) Slope. (**b**) Land use type. (**c**) Distance to infrastructure. (**d**) Distance to digital footprint. (**e**) Distance to road. (**f**) Distance to recreational resource.

This study created layers for each resistance factor in ArcGIS based on the evaluation criteria and their respective weights (Fig. 15).

**Fig. 16:**
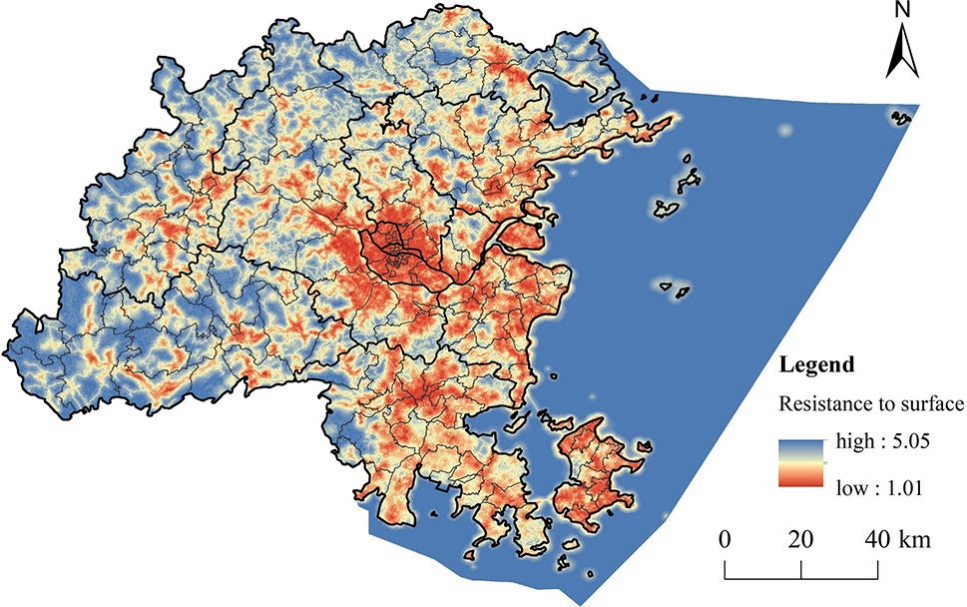
Comprehensive RRS. (created by ArcMap, version 10.5, http://www.esri.com/)

A weighted sum of these factors was calculated using the raster calculator, leading to the comprehensive RRS for Fuzhou City (Fig. 16). The resistance values for recreational activities in Fuzhou show a trend of higher values in the eastern part and lower values in the west. The areas with high resistance are mainly found in the central and eastern core regions of Fuzhou, which are characterized by land cover types such as cropland and construction land, featuring concentrated development, extensive transportation networks, and a high density and variety of recreational activities. Although there are some high-resistance areas along roads in the western regions, they are smaller and more scattered. Conversely, low-resistance areas are primarily located in the mountainous regions in the west and north of Fuzhou, where there is significant topographic variation, abundant forest resources, limited transportation access, and lower levels of human development.

**Fig. 17:**
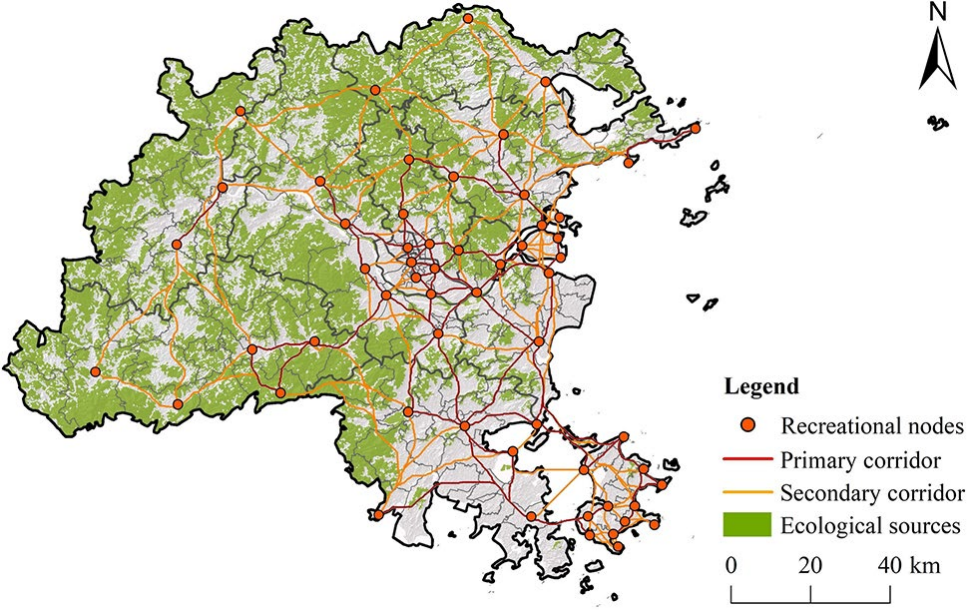
Recreational corridors. (created by ArcMap, version 10.5, http://www.esri.com/).

Using the Linkage Mapper tool, 165 RCs were simulated, covering a total of 3795.21 km, which includes 64 primary corridors and 101 secondary corridors (Fig. 17). These corridors are mainly concentrated in the central urban areas and eastern coastal regions of Fuzhou, which are relatively flat, have high transportation accessibility, and are primarily designated for construction, with frequent human activity. However, due to the dense distribution of RNs in these areas, the corridors tend to be relatively short.

### Reconstruction of ecological security pattern

**Fig. 18:**
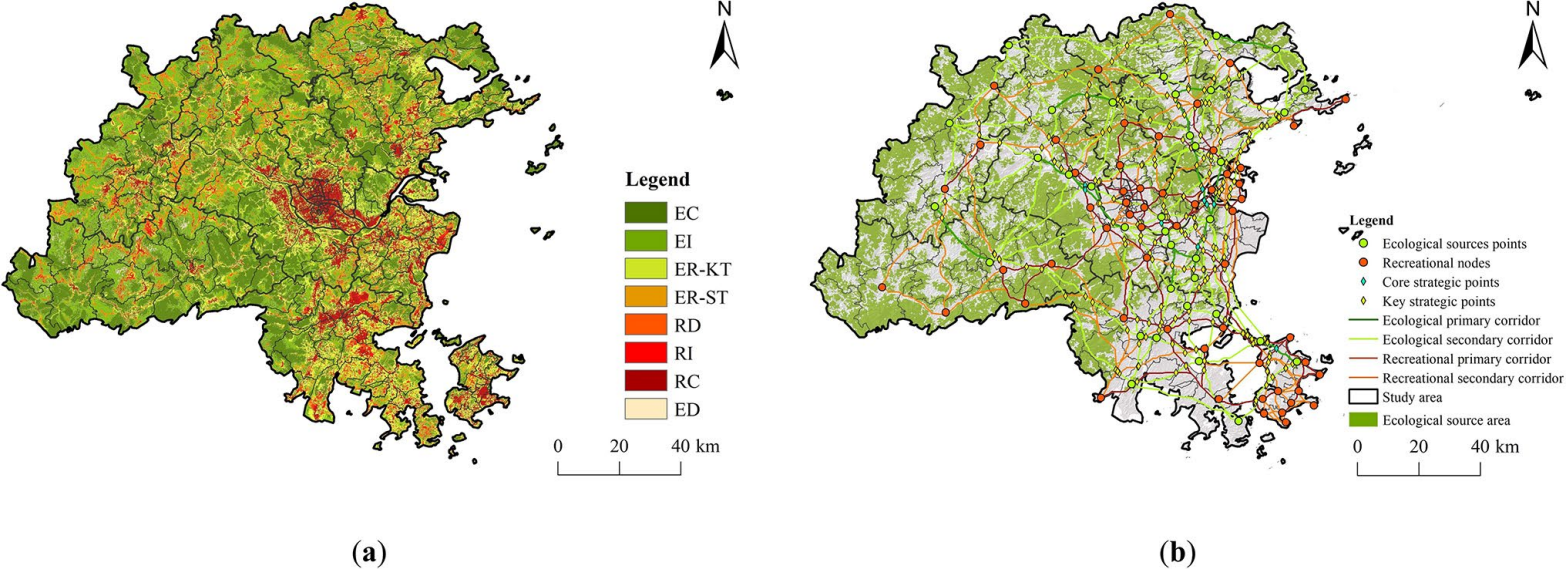
Reconstruction of ESP elements. (created by ArcMap, version 10.5, http://www.esri.com/) (**a**) Ecological and recreation function trade-off zone; (**b**) Spatial element structure of ESP.

Based on the results of the ERS and RRS analyses, along with the previously developed ecological-recreational function trade-off matrix (Fig. 3), the ESP zoning results for Fuzhou City were illustrated through a raster calculator (Fig. 18). The ECZ occupies the largest area at 4319.82 km², representing 36% of Fuzhou City’s total land. The EIZ zone follows with an area of 3265.79 km², making up 27% of the study area, primarily located in the mountainous regions to the west and north parts of Fuzhou City. The areas of the ER-KTZ, RDZ, RCZ, ER-STZ, EDZ, and RIZ decrease in size in that order. By overlaying the ECs and RCs, this study identified 494 strategic points (Fig. 18). Among these, 19 core strategic points are located in the southeastern part of Cangshan District, the eastern part of Mawei District, the central part of Changle District, the southern part of Lianjiang County, the eastern part of Minhou County, and the northern part of Pingtan County, mainly along the Minjiang River in central Fuzhou City. Additionally, 475 key strategic points were identified, with Lianjiang County, Changle District, Pingtan County, and Minhou County having a higher concentration of these points, indicating active ecological and recreational processes and potential conflicts, which require improved management and regulation of ecological protection and recreational activities.

**Fig. 19:**
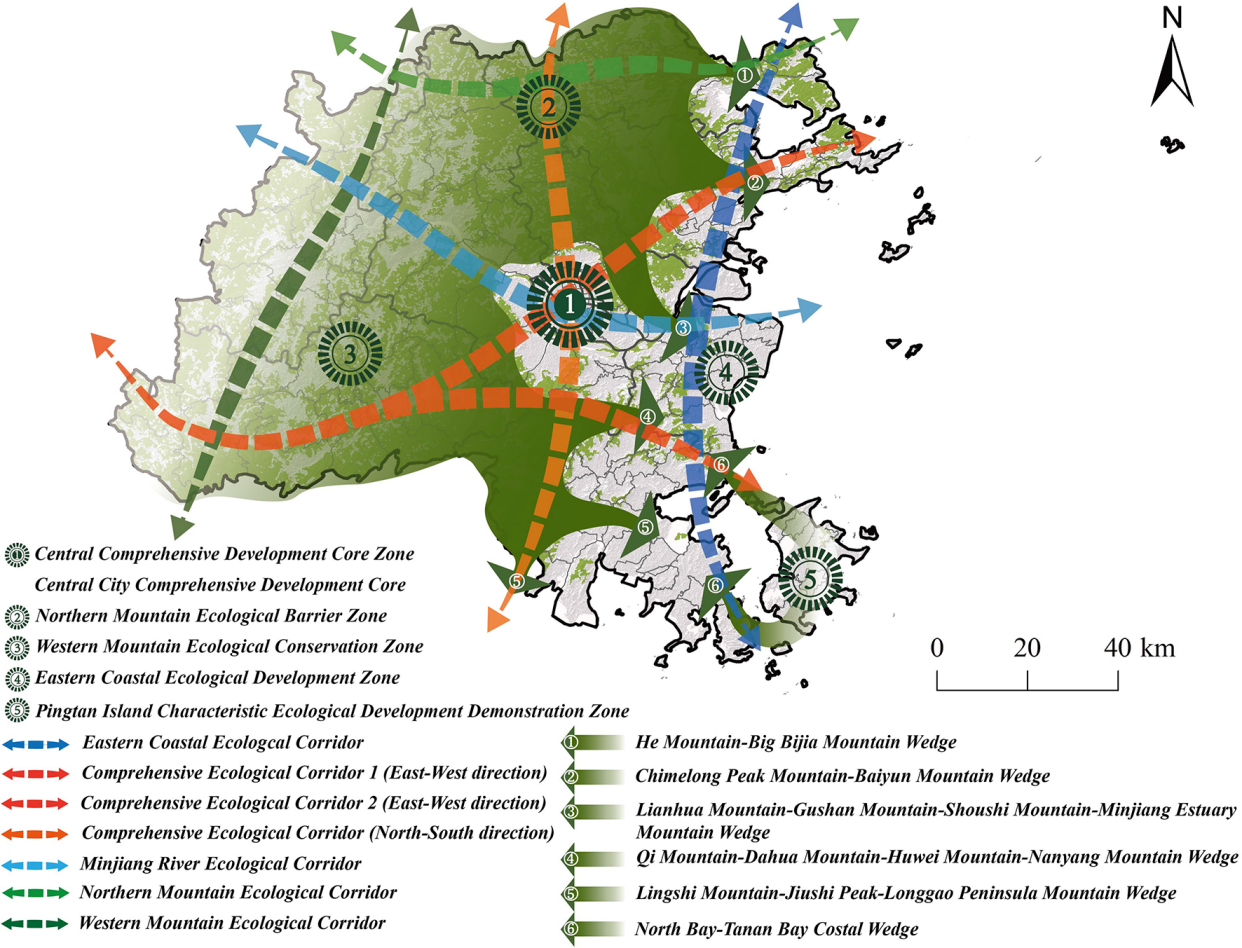
Reconstruction of ESP in Fuzhou City.

Based on the overall findings from the ESP zoning, ECs construction, and strategic point identification, this study proposes an optimization strategy for Fuzhou City’s ecological security pattern, referred to as “one core, five zones, six corridors, and seven wedges” (Fig. 19). The “one core” represents the Central Urban Comprehensive Development Core. The five zones consist of the Central Comprehensive Development Core Zone, the Eastern Coastal Ecological Development Zone, and Pingtan Island Characteristic Ecological Development Demonstration Zone, Western Mountain Ecological Conservation Zone and Northern Mountain Ecological Barrier Zone. The six corridors include the Comprehensive Ecological Corridor running east-west and north-south, the Minjiang River Ecological Corridor, the Eastern Coastal Ecologal Corridor, the Western Mountain Ecological Corridor, and the Northern Mountain Ecological Corridor. The seven wedges comprise the He Mountain - Big Bijia Mountain Corridor, the Chimelong Peak Mountain - Baiyun Mountain Corridor, the Lianhua Mountain - Gushan Mountain - Shoushi Mountain - Minjiang Estuary Mountain Corridor, the Qi Mountain - Dahua Mountain - Huwei Mountain - Nanyang Mountain Corridor, the Lingshi Mountain-.Jiushi Peak - Longgao Peninsula Mountain Corridor, the Lingshi Mountain- Jiangyin Mountain Corridor, and the North Bay - Tanan Bay Costal Eorridor.

## Discussion

### Applicability and advantages of multifunctional balance in the construction of ESP

The development of a multifunctional ESP is intended to enhance land spatial configurations, stabilize ecosystems, and facilitate high-quality regional development^11,30^. Existing research on ESP predominantly emphasizes the establishment of single-function networks focused on ecological protection, primarily constructing ESP through the application of MSPA and MCR models^15^. However, this research trajectory tends to overlook other types of corridors, such as those designated for recreation and hydrological protection^46^, thereby neglecting the significance of multi-objective and multi-dimensional collaborative management within ecosystems. It particularly fails to acknowledge the essential role of recreational functions in sustaining ecological balance and fostering a harmonious relationship between humans and nature^46^. As a result, the potential of ESP to promote this harmonious coexistence, enhance public ecological awareness and recreational benefits, and achieve sustainable development objectives remains underexploited, leading to a lack of comprehensiveness and sustainability in the formulation of ecological security patterns. Fuzhou City’s diverse natural environment and recreational spaces constitute a complex and mosaic ESP. Although the extent area of ESAs is considerable, there are notable shortcomings, including spatial fragmentation and inadequate connectivity^41^. Concurrently, the growing diversity and demand for recreational activities present challenges for effective ecosystem management, thereby complicating the achievement of the ecosystem’s overall benefits. Consequently, it is necessary to strike a balance between ecological protection and recreational development, guided by ecological principles. Consequently, this research develops an ESP framework that considers the integrated development of ecological systems and recreational activities. This approach offers a more rigorous and holistic analytical methodology for harmonizing ecological conservation with recreational development in urban environments.

In previous research, the construction of multifunctional ESP primarily focused on the extraction of multifunctional ESs^30,46^, lacking in-depth analysis and comprehensive coordination of the relationships between different functions, especially regarding the coupling mechanisms between ecological and recreational functions. This study constructed eight functional zones using a trade-off matrix, highlighting the complexity of the interconnections in the coupling process of ecological and recreational multifunctionality, which is similar to the findings of Liu et al.^52^. By balancing ecological and recreational functions, this research delves into the coupling mechanisms and interactions between multifunctional ESP, allowing for a more accurate and reasonable determination of multifunctional spatial zoning. This, in turn, facilitates the formulation of more effective ecological protection measures, providing viable solutions for urban ecological environment protection and recreational development, and making the construction of ecosystems at the urban scale more flexible and practical. This approach forms the foundation and key component of research on multifunctional ESP, providing a scientific basis and important criteria for developing strategies that ensure regional sustainable development.

To enhance the scientific rigor in the development of RSP, this study employs a comprehensive approach that integrates recreational flow analysis with circuit theory to identify RNs and delineate RCs. Recreational flow analysis addresses various developmental challenges, including the equitable utilization of recreational resources, the design and management of recreational spaces, and the preferences exhibited in recreational behavior^33^. This analysis is pivotal in optimizing the distribution of RRs and enhancing the quality of recreational experiences. However, its application within the realm of ecological protection remains relatively limited. In contrast to methodologies that investigate ecological security patterns based solely on individual ecological functions, this study adopts a synergistic approach that combines ArcGIS spatial analysis with recreational flow analysis. This integration enhances the comprehensiveness and scientific validity of constructing ecological security patterns, making it more adept at addressing the multifunctional and multi-scenario development requirements of urban ecological spaces^35^. Furthermore, this research proposes specific development strategies informed by the functional zoning of ESP. These strategies aim to foster a harmonious coexistence between recreational activities and ecological spaces while safeguarding ecosystem functions, thereby facilitating effective land use management and promoting sustainable development.

### Planning and control strategy for ESP

This study reconstructs the ESP using a tradeoff matrix, identifying eight major functional zone types and 494 strategic points, along with corresponding planning and management strategies.

Specifically, the ECZ is highlighted as the primary distribution area for Fuzhou’s ecological sources and is vital to the ecosystem. It is crucial to establish clear ecological protection boundaries, restore vegetation, safeguard natural water systems, control soil erosion, and maintain healthy ecosystem circulation. The EIZ should prioritize ecological protection while fostering coordinated regional development and promoting green, low-carbon economic growth. Enhancing the ecosystem’s economic value can be achieved through industrial transformation, upgrading, and resource recycling.

The RCZ, as the core of various districts and counties, should utilize its rich recreational resources, extensive market, and established industrial framework to offer high-quality recreational experiences for both residents and tourists, thereby stimulating the development of nearby recreational areas. The RIZ, which is characterized by a scattered spatial layout and primarily agricultural land use, should incorporate recreational functions from Fuzhou’s urban centers and extend its services to eastern coastal and western mountainous regions. The RDZ should create diverse ecological recreational areas to connect and integrate with surrounding regions, ensuring the ecosystem and landscape remain intact and continuous.

The ER-KTZ must focus prioritize the balance between ecological and recreational functions while rigorously protecting thebalance between ecological and recreational functions while rigorously protecting the ecological environment. This includes delineating suitable areas for recreational activities, allowing for small-scale recreation and ecotourism. The ER-STZ needs to provide sufficient recreational spaces and opportunities for the public while maintaining the balance between ecological and recreational functions, with the establishment of basic recreational infrastructure, like greenways. The EDZ is relatively small, it suffers from significant soil erosion and lacks recreational resources. Measures should be taken to address soil erosion through afforestation, converting farmland back to forests and grasslands, promoting ecological restoration and protection, and improving transportation infrastructure to support eco-agriculture development in the western and northern mountainous regions.

The Central Urban Comprehensive Development Core, serving as the urban nucleus of Fuzhou City, must prioritize the optimizing of the urban ecosystem to enhance living conditions and improve the quality of recreational spaces. The principal aims include augmenting the overall efficacy of urban ecological services, expanding both the scale and quality of recreational space development, and furtherstrengthening the network of ECs within the central urban area, thereby fostering a more livable and enjoyable urban ecological framework. Among the five designated zones, the Central Comprehensive Development Core Zone is tasked with capitalizing on its central position to propel the advancement of urban recreational infrastructure, elevate urban quality, and cultivate an ecological environment that is conducive to livability, business, and recreation. The Eastern Coastal Ecological Development Zone should prioritize the reinforcement of marine ecological protection, the implementation of sustainable utilization strategies for marine fishery resources, and the safeguarding of the nearshore marine ecological environment. The Pingtan Island Characteristic Ecological Development Demonstration Zone should adhere to principles of green development, emphasizing island ecological protection and restoration while promoting island tourism and marine culture industries. The Western Mountain Ecological Conservation Zone must focus on enhancing ecological protection and restoration efforts in mountainous areas, maintaining biodiversity, prioritizing ecological strategies, and actively developing ecological agriculture and tourism, alongside improving rural environmental management and preserving a healthy mountain-water ecosystem. The Northern Mountain Ecological Barrier Zone should concentrate on protecting water sources and establishing ecological security barriers, instituting an ecological compensation mechanism, and moderately advancing eco-tourism and other green industries. As a city characterized by its connection to both mountains and seas, Fuzhou must strengthen the communication and inter linkages between these two landscapes, thereby creating ecological corridors that bridge the mountains and the sea, with strict restrictions on development activities within these corridors. By promoting the integration and optimized configuration of ecological and recreational resources along the six corridors and implementing a strategy for integrated mountain-sea protection, Fuzhou can facilitate coordinated development between its mountainous and coastal areas. Furthermore, Fuzhou should enhance the protection of natural resources in the seven wedge-shaped areas to mitigate over development and pollution, while simultaneously leveraging the natural conditions in these areas to cultivate diverse green recreational spaces, including parks, green areas, and ECs.

### Limitations and prospects for future research

This research integrates recreational functions into the development of the ESP, thereby establishing a novel framework that reflects the intricate internal dynamics of the coupled ecological-recreation system while constructing an ESP that encompasses both ecological and recreational functions. Nonetheless, the study is constrained by certain limitations, primarily due to the substantial workload associated with data collection, processing, and analysis.

In the formulation of ERS and RRS, there is a need for further refinement and expansion of the indicator system. The selection of resistance factors and the allocation of weight values have a significant impact on the ESP^15^. Currently, there are no standardized criteria, either domestically or internationally, for the selection of these factors, And many studies rely on prior research, which introduces a level of subjectivity. Consequently, future investigations should consider the inclusion of additional indicators, such as habitat quality^53^, nighttime light data^54^, and policy factors^55^ to enhance the scientific rigor of the study and improve the accuracy of ESP construction.

While this study examines recreational flow in Fuzhou, the data available is limited in both quantity and scope. It lacks comprehensive information regarding visitor demographics, including age, gender, and occupation, as well as their recreational motivations^33^. Future research should aim to broaden data collection through multiple sources, such as mobile signaling and trajectory information, to more thoroughly elucidate the underlying patterns and external influences affecting recreational flow in Fuzhou.

For the construction of ECs and RCs, this study primarily employed the Linkage Mapper tool based on circuit theory for identification and classification purposes. Future research could enhance the identification of corridors by integrating additional methods, such as the gravity model^56^ and spatial syntax^11^, thereby facilitating the construction of more targeted ECs and RCs.

## Conclusions

This research examines Fuzhou City and, focusing on the integrated development of ecological and recreational spaces, and proposes a framework for constructing ESP that emphasizes both ecological and recreational functions. The study aims to optimize the ESP by taking into account the existing ecological environment and planning development strategies specific to Fuzhou. The key findings are as follows: a total of 36 ESAs were identified, encompassing an area of 5807.90 km², which represents 48.53% of the total land area of Fuzhou City. While these ESAs are primarily situated in the western and northern parts of the city, their distribution is relatively sparse. Additionally, 98 ECs were delineated, with an aggregate length of 2500.55 km, utilizing circuit theory. The research also identified 100 EPPs and 146 EBPs. Through the application of ArcGIS spatial analysis and recreation flow analysis, 57 RNs were identified, predominantly located in Gulou District, Taijiang District, Cangshan District, Mawei District, Pingtan County, and Lianjiang County, revealing a pattern of dense distribution in the east region and sparse presence in the west. Furthermore, 165 RCs were identified, totaling 3795.21 km in length. Both the ESPs and RNs exhibit notable spatial distribution disparities between the eastern and western regions. The establishment of ERS and RRS addresses the deficiencies in multi-factor considerations, thereby better aligning with the integrated development of ecological and recreational functions. The development of ECs and RCS enhances connectivity and interaction between the eastern and western regions, facilitating ecological linkages, optimizing resource allocation, and promoting regional coordination. The study identified a total of 494 strategic points, comprising 19 core strategic points and 475 key points, primarily concentrated in the central and eastern region of Fuzhou, where there is a pressing need for enhanced environmental protection and recreational management. Based on the resistance surfaces associated with ecological and recreational functions, a tradeoff matrix was constructed, leading to the classification of eight functional zones. Customized planning and development recommendations were formulated for each zone. In alignment with Fuzhou’s territorial spatial planning and related research, this study proposes a strategy for reconstructing the ESP characterized by “one core, five zones, six corridors, and seven wedges.” By integrating recreational functions into ecological construction, the research contributes to the systematic and scientific advancement of the ESP, providing significant insights for the coordinated development of urban ecological and recreational spaces. The methods and results of this study, which integrates ecological and recreational functions to establish an ecological security pattern, may offer valuable guidance for other regions experiencing similar challenges related to urbanization and environmental degradation. These findings can inform strategies for ecological land use planning, recreational development, the coordinated advancement of urban ecological and recreational spaces, and the formulation of ecological protection policies.

## Data Availability

The datasets used and analyzed during the current study available from the corresponding author on reasonablerequest.
